# The impacts of e‐cigarette flavours: An overview of systematic reviews

**DOI:** 10.1111/add.70017

**Published:** 2025-02-25

**Authors:** Jonathan Livingstone‐Banks, Nargiz Travis, Monserrat Conde, Yixian (Crystal) Chen, Padmo Zi, Holly Jarman, Nicola Lindson, Jamie Hartmann‐Boyce

**Affiliations:** ^1^ Nuffield Department of Primary Care Health Sciences University of Oxford UK; ^2^ Lombardi Comprehensive Cancer Center Georgetown Medical University Washington D.C. USA; ^3^ Department of Integrative Oncology British Columbia Cancer Research Institute Vancouver Canada; ^4^ Department of Epidemiology University of Michigan School of Public Health Ann Arbor MI USA; ^5^ Department of Health Management and Policy University of Michigan School of Public Health Ann Arbor MI USA; ^6^ Department of Health Promotion and Policy University of Massachusetts Amherst Amherst MA USA

## Abstract

**Background and Aims:**

E‐cigarette flavours have the potential to impact the appeal, harms and use of e‐cigarettes and combustible tobacco. Systematic reviews have synthesised evidence on their impacts but have always focused on specific outcomes or populations. This overview aimed to draw together syntheses from past systematic reviews of e‐cigarette flavours to provide a holistic, population‐wide view.

**Methods:**

Overview of systematic reviews investigating the impacts of e‐cigarette flavours on any outcome. We searched six databases to February 2024, and appraised reviews using AMSTAR2. We used association direction plots and narratively synthesised results.

**Results:**

We included 32 reviews (11 higher quality; 21 lower). Reviews reported impacts of e‐cigarette flavours on: appeal/perceptions of vaping (13 reviews); harms (12); smoking (7); and vaping (13) behaviours. Availability of non‐tobacco e‐cigarette flavours may increase the appeal of (8 reviews) and motivation to try/continue using e‐cigarettes (5) and decrease harm perceptions (5). There were no clear differences in impacts based on age or history of combustible tobacco use, and little difference in findings between higher and lower quality reviews. Two reviews indicated that among adolescents, experimenting with different flavours increased e‐cigarette appeal. Twelve reviews indicated that a range of specific flavours (including cinnamon, menthol and various sweet/fruity flavours) may be harmful; this often came from in vitro experiments and chemical analyses. Findings were inconclusive on the impact of e‐cigarette flavours on smoking cessation (six reviews not showing clear impact), smoking initiation (two reviews not showing clear impact) and vaping initiation (two reviews showing increased initiation and two not showing clear impact).

**Conclusions:**

Non‐tobacco flavourings for e‐cigarettes may increase e‐cigarette appeal and harms; this increase may vary by flavour and apply across different population groups. The impacts of e‐cigarette flavours on e‐cigarette and cigarette use are inconclusive.

## INTRODUCTION

There is high certainty evidence that electronic cigarettes (e‐cigarettes) with nicotine can help people stop smoking [1]. However, young people who do not smoke also use e‐cigarettes [2]. Although considerably less harmful than combustible tobacco [3, 4], e‐cigarettes are unlikely to be harm‐free, and hence, their use in people who do not smoke is likely to introduce some level of health risk [5]. As a result, policymakers often grapple with how to encourage people who smoke to switch to vaping, while discouraging people who do not smoke (particularly young people) from vaping. E‐cigarettes are not a uniform product class, they can vary by characteristics including device type, e‐liquid content and flavourings [6]. These variations can impact their use by people who smoke and by people who do not smoke, as well as their potential safety profile [7, 8, 9].

Flavours are a topic of particular interest and of policy and research focus. Questions persist regarding the role of e‐cigarette flavours in vaping and smoking behaviours, with many jurisdictions considering or already having enacted restrictions on certain flavours in electronic cigarettes [10]. It has been argued that flavours may be an inducement to vaping in young people [11], but also that they may encourage adults who smoke to switch to a reduced harm product [4]. Numerous systematic reviews have been published in this area, covering dimensions including youth uptake of vaping, appeal of vaping (in both young people and adults) and use of e‐cigarettes by adults wishing to switch from smoking to vaping (e.g. Notely *et al*., [11] Effah *et al*., [12] Gades *et al*. [13] and Zaher *et al*. [14]). However, each focuses on a specific area, and not the intersection of these different domains. This can make it challenging to assess the multiple possible effects of e‐cigarette flavours across different populations and outcomes.

To enable a holistic view of the extant literature on e‐cigarette flavours, we set out to conduct an overview of systematic reviews to:

- Catalogue and signpost the existing evidence synthesis literature on this topic
- Evaluate the strengths and weaknesses of evidence in this space, including the quality of existing systematic reviews
- Synthesize existing literature on both youth and adults, to discuss similarities and differences in the literature

## METHODS

We reported our work according to guidelines for the Preferred Reporting Items for Systematic Reviews and Meta‐Analyses (PRISMA) [15]. We pre‐registered a protocol on Open Science Framework: https://osf.io/cx9n8. We were unable to conduct proposed data charting and visualisation based on appeal/use measures, divided into adults and children, because of insufficient data, but there were no other deviations from protocol.

### Inclusion criteria

Eligibility was determined based on, intervention/exposure: flavoured e‐liquid in e‐cigarettes (we considered any flavour information as categorised by authors); study type: peer reviewed and published systematic reviews defined according to the existence of the following characteristics: (a) research question, (b) reproducible search strategy including search databases, (c) screening methods and inclusion and exclusion criteria, (d) quality appraisal methods, and (e) reporting of data synthesis [16]. We did not exclude reviews on the basis of participants or outcomes, although we anticipated outcomes of interest would include (but not be limited to): uptake and appeal of vaping; vaping behaviours; cigarette smoking behaviours; use of other commercial tobacco; and nicotine products. Reviews were considered regardless of whether they covered the same topic as another included review.

### Searches and study selection

An information specialist designed and conducted systematic searches in MEDLINE, Embase, PsycINFO, Cochrane Database of Systematic Reviews, PROSPERO and Epistomonikos using terms related to systematic reviews, e‐cigarettes and flavours from 2004 (when e‐cigarettes appeared on the global market) to 13 February 2024. Results were imported into Covidence and screened in duplicate independently by two reviewers with discrepancies resolved by discussion or through referral to a third author. An example search strategy is listed in the supplementary material.

### Data extraction and critical appraisal

Data extraction and critical appraisal were conducted by one reviewer using a pre‐defined and piloted data extraction form and checked by a second reviewer with discrepancies resolved through discussion or referral to a third reviewer. We assessed the quality of each included systematic review using the critical domains from A Measurement Tool to Assess Systematic Reviews‐2 (AMSTAR‐2) checklist [17]. Each review was assigned a rating of ‘higher’ or ‘lower’ quality based on whether they had received answers of ‘yes’ or ‘partial yes’ to at least six of AMSTAR‐2's seven critical domains, as per the methods of Hartmann‐Boyce *et al*. [18]. These critical domains included a range of criteria; namely, the existence of and adherence to a protocol, the use of a comprehensive search strategy, the provision of a justified list of excluded studies, the use of a satisfactory risk of bias assessment technique, the use of appropriate meta‐analytical methods (where meta‐analysis was conducted), the consideration of risk of bias in discussing the results of the review and the adequate investigation of publication bias where appropriate. Reviews which were deemed of lower quality were not excluded from the overview of reviews, but their appraisal was accounted for when considering their results. We recorded the number of reviews and within them the number of studies supported by industry funding, where reported and report these separately.

### Data synthesis and analysis

For each study, we narratively synthesized information on the included studies and the conclusions of the review. We noted where studies were in multiple reviews to highlight potential overlap. For quantitative reviews, where present, we presented pooled estimates, 95% CI, and heterogeneity values where they were given, as well as the results of critical appraisal and any judgements of certainty provided by the authors [e.g. Grading of Recommendations Assessment Development and Evaluation (GRADE) ratings]. For qualitative reviews, we presented key themes as identified by study authors as well as results of critical appraisal. Findings were grouped by outcome and within that by population. Where more than two reviews contributed data to an outcome, this information was charted in effect/association direction plots, following synthesis without meta‐analysis guidance [19].

## RESULTS

**FIGURE 1:**
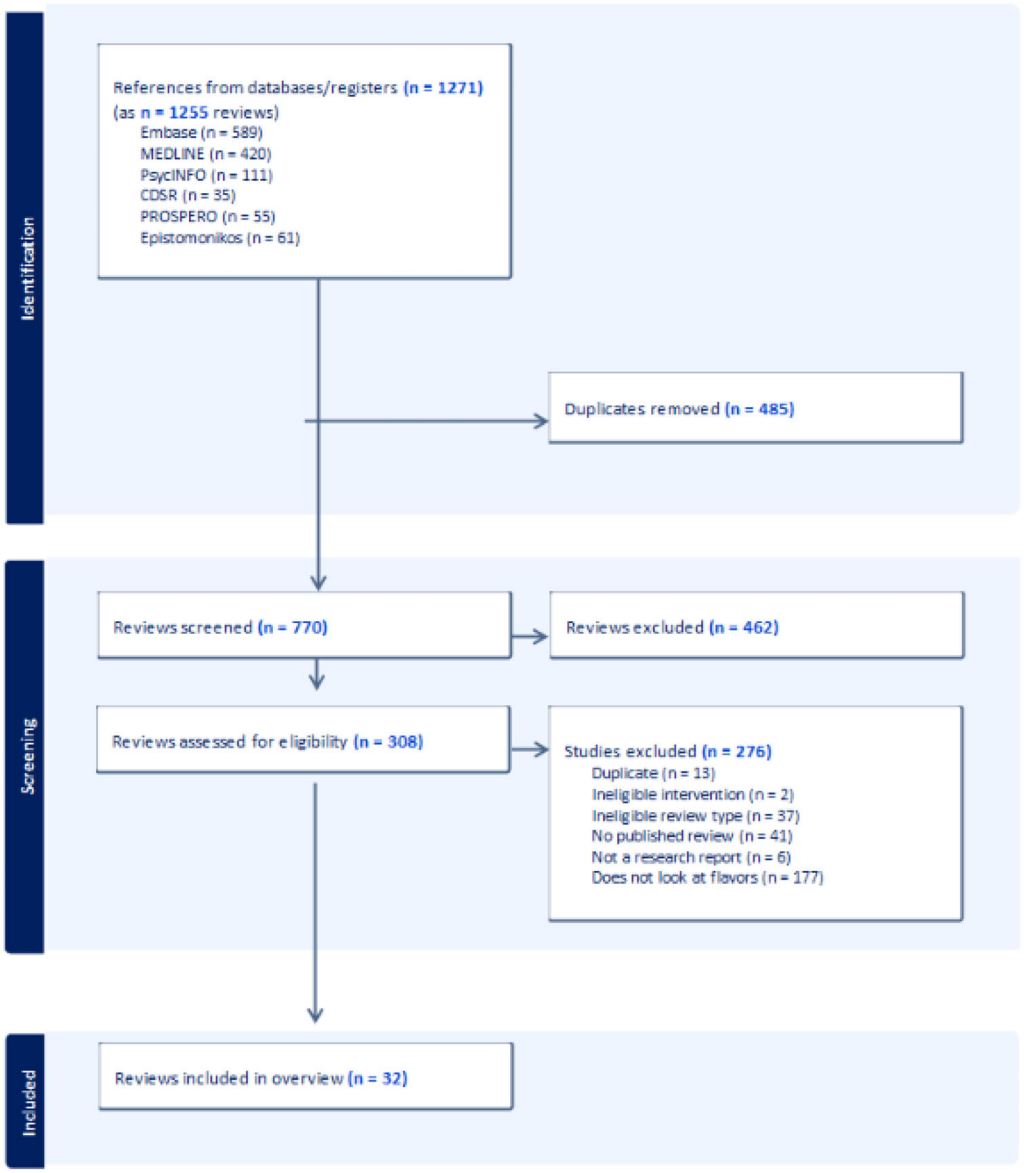
Preferred Reporting Items for Systematic Reviews and Meta‐Analyses (PRISMA) flow diagram.

Our searches found 770 records after deduplication, 462 of which we deem irrelevant based on title and abstract screening. We reviewed full texts of the remaining 308 records and excluded a further 276. Figure 1 documents study flow and reasons for exclusion.

### Characteristics of included reviews

We included 32 reviews including 1967 primary studies (including some studies that feature in multiple reviews), of which five were qualitative evidence syntheses and one was mixed methods (Table 1). In the included reviews, we identified evidence relating to the impacts of e‐cigarette flavours from 326 unique primary studies. Of these, 33 were included in more than one review. Reviews ranged in size from including six to 400 studies, covering 804 to 106 575 participants, although only seven reviews clearly reported the number of participants covered. Latest search dates for reviews ranged from 2013 up to mid‐2023. Seven investigated flavours as a primary focus and one as a secondary focus. Remaining reviews were not focused on flavours at all, but reported incidental findings relating to the impact of flavours. Eight reviews focussed specifically on younger populations, two on pregnancy, one of vulnerable populations and one on women who use hormonal contraceptives. Reviews investigated a range of outcomes relating to the impact of e‐cigarette flavours, including impacts on appeal or perceptions of vaping, harms from vaping and impacts on combustible tobacco or e‐cigarette use behaviour. No reviews reported receiving funding from the tobacco or e‐cigarette industry or declared any financial conflicts of interest pertaining to the review in question. Included studies with industry funding, where reported by reviews, are reported in Table 1.

**TABLE 1:** Characteristics of included reviews.

Study ID	Evidence types included	No. of included studies	No. of included studies funded by industry	No. of included participants	Latest search date	Were flavours a focus?	Overall AMSTAR‐2 rating	Outcomes reported
Ahuja *et al*. 2023 [20]	Human observational	8	Not reported	Not reported	December 2021	Incidental findings	Lower quality	Appeal/perceptions of vaping
Banks *et al*. 2023 [21]	Human experimental; human observational; case studies	400	Not reported in aggregate	Not reported	July 2020	Incidental findings	Lower quality	Harms from vaping, vaping behaviours
Calder *et al*. 2021 [22]	Human observational; qualitative; human experimental	23	Not reported	Not reported	February 2020	Incidental findings	Higher quality	Vaping behaviours
Camoni *et al*. 2023 [23]	In vitro experimental	84	Not reported	Not reported	August 2023	Secondary focus	Higher quality	Harms from vaping
Campbell *et al*. 2020 [24]	Qualitative	21	1	497	February 2019	Incidental findings	Higher quality	Appeal/perceptions of vaping
Ciapponi *et al*. 2021 [25]	Human observational; human experimental	52	Not reported	Not reported	August 2019	Incidental findings	Lower quality	Tobacco use behaviours
Dautzenberg *et al*. 2023 [26]	Human observational	22	Not reported	106 575	March 2023	Incidental findings	Lower quality	Vaping behaviours
Dyson *et al*. 2022 [27]	Qualitative; human experimental	10	Not reported	19 028	March 2021	Primary focus	Lower quality	Vaping behaviours
Erku *et al*. 2020 [28]	Human observational; qualitative	45	Not reported	13 548	September 2018	Incidental findings	Lower quality	Appeal/perceptions of vaping
Feirman *et al*. 2016 [29]	Human observational; qualitative; human experimental	32	Not reported	Not reported	September 2013	Primary focus	Lower quality	Appeal/perceptions of vaping, vaping behaviours
Gades *et al*. 2022 [13]	Human experimental; human observational; animal experimental	104	Not reported	Not reported	August 2020	Primary focus	Lower quality	Appeal/perception of vaping
Gentry *et al*. 2019 [30]	Human observational; qualitative; human experimental	9	Not reported	Not reported	March 2017	Incidental findings	Higher quality	Appeal/perceptions of vaping
Han *et al*. 2022 [31]	Human observational; qualitative; human experimental	85	Not reported	Not reported	March 2022	Incidental findings	Lower quality	Vaping behaviours
Hod *et al*. 2022 [32]	Human observational; in vitro experimental; animal experimental	13	Not reported	Not reported	December 2020	Incidental findings	Higher quality	Vaping behaviours
Kinouani *et al*. 2020 [33]	Human observational; qualitative	6	Not reported	Not reported	March 2018	Incidental findings	Lower quality	Appeal/perceptions of vaping
Kowitt *et al*. 2017 [34]	Qualitative	20	Not reported	Not reported	April 2016	Primary focus	Lower quality	Appeal/perceptions of vaping, harms from vaping
Larue *et al*. 2021 [35]	Human experimental	45	Not reported	919	May 2021	Incidental findings	Lower quality	Harms from vaping
Liber *et al*. 2023 [36]	Human observational	29	4	Not reported	May 2022	Primary focus	Higher quality	Tobacco use behaviours
Lindson *et al*. 2024 [1, 37, 38]	Human experimental	88	14	27 235	February 2024	Incidental findings	Higher quality	Harms from vaping, tobacco use behaviours, vaping behaviours
McNeill *et al*. 2022 [9]	Human experimental; human observational; in vitro experimental; animal experimental; case studies	28	9	804	July 2021	Primary focus	Higher quality	Appeal/perceptions of vaping, harms from vaping, vaping behaviours
Meernik *et al*. 2019 [39]	Human observational; human experimental	51	3	Not reported	March 2018	Primary focus	Lower quality	Appeal/perceptions of vaping, tobacco use behaviours, vaping behaviours
Notley *et al*. 2022 [11]	Human observational; qualitative; human experimental	58	Not reported	Not reported	September 2020	Primary focus	Higher quality	Appeal/perceptions of vaping, harms from vaping, tobacco use behaviours, vaping behaviours
Novelli *et al*. 2022 [40]	Human experimental; in vitro experimental	38	Not reported	Not reported	October 2020	Incidental findings	Lower quality	Harms from vaping
Pepper and Brewer 2014 [41]	Human observational; qualitative; human experimental; in vitro experimental	49	6	Not reported	July 2013	Incidental findings	Lower quality	Appeal/perceptions of vaping
Riley *et al*. 2016 [42]	Human experimental; human observational; case studies	13	3	Not reported	June 2015	Incidental findings	Lower quality	Harms from vaping
Sharma *et al*. 2021 [43]	Human observational; qualitative; human experimental	25	Not reported	Not reported	April 2019	Incidental findings	Lower quality	Appeal/perceptions of vaping
Ward *et al*. 2020 [44]	Chemical analyses	92	Not reported	Not reported	May 2020	Incidental findings	Lower quality	Harms from vaping
Wilson *et al*. 2022 [45]	In vitro experimental; human experimental	18	2 identified in report	Not reported	December 2020	Incidental findings	Lower quality	Harms from vaping
Yan *et al*. 2023 [46]	Human observational	48	Not reported	Not reported	June 2022	Incidental findings	Higher quality	Other
Yang *et al*. 2020 [47]	Human observational; in vitro experimental; human survey	99	Not reported	Not reported	December 2019	Incidental findings	Lower quality	Harms from vaping
Yoong *et al*. 2021a [48]	Human observational	327	Not reported	Not reported	August 2020	Incidental findings	Lower quality	Vaping behaviours
Yoong *et al*. 2021b [49]	Human observational	25	0	Not reported	September 2020	Incidental findings	Higher quality	Tobacco use behaviours, vaping behaviours

Abbreviations: AMSTAR‐2, A Measurement Tool to Assess Systematic Reviews‐2.

### Quality of included reviews

We deemed 11 reviews to be of higher quality and 21 reviews to be of lower quality (Table 2). The critical domains most commonly assessed as ‘no’ were not listing excluded studies along with reasons for exclusion, not citing a pre‐registered protocol that provided sufficient detail on methods, and not conducting (or providing enough detailed reporting of) quality appraisal of included studies.

**TABLE 2:** AMSTAR‐2 summary.

Review	Protocol	Searches	Exclusions	Risk of bias	Meta‐analysis methods	Bias in results	Publication bias	Overall rating
Ahuja *et al*. 2023 [20]	No	Partial yes	No	Yes	N/A	No	N/A	Lower quality
Banks *et al*. 2023 [21]	Yes	Partial yes	No	No	Yes	Yes	Yes	Lower quality
Calder *et al*. 2021 [22]	Yes	Partial yes	No	Yes	N/A	Yes	N/A	Higher quality
Camoni *et al*. 2023 [23]	Partial yes	Partial yes	Yes	Yes	Yes	Yes	No	Higher quality
Campbell *et al*. 2020 [24]	Yes	Partial yes	Yes	Yes	N/A	Yes	N/A	Higher quality
Ciapponi *et al*. 2021 [25]	Yes	Partial yes	No	Partial yes	No	No	No	Lower quality
Dautzenberg *et al*. 2023 [26]	Yes	No	No	No	N/A	Yes	N/A	Lower quality
Dyson *et al*. 2022 [27]	No	Partial yes	No	Partial yes	N/A	No	N/A	Lower quality
Erku *et al*. 2020 [28]	Partial yes	Partial yes	No	Partial yes	N/A	No	N/A	Lower quality
Feirman *et al*. 2016 [29]	No	Yes	No	Yes	N/A	No	N/A	Lower quality
Gades *et al*. 2022 [13]	No	Partial yes	No	Yes	N/A	No	N/A	Lower quality
Gentry *et al*. 2019 [30]	Yes	Partial yes	No	Partial yes	N/A	Yes	N/A	Higher quality
Han *et al*. 2022 [31]	Partial yes	Yes	No	Yes	N/A	No	N/A	Lower quality
Hod *et al*. 2022 [32]	Partial yes	Partial yes	No	Yes	N/A	Yes	N/A	Higher quality
Kinouani *et al*. 2020 [33]	No	Partial yes	No	No	N/A	No	N/A	Lower quality
Kowitt *et al*. 2017 [34]	No	Partial yes	No	Yes	N/A	No	N/A	Lower quality
Larue *et al*. 2021 [35]	No	Partial yes	No	Yes	Yes	Yes	Yes	Lower quality
Liber *et al*. 2023 [36]	No	Partial yes	Yes	Yes	N/A	Yes	N/A	Higher quality
Lindson *et al*. 2024 [1]	Yes	Yes	Yes	Yes	Yes	Yes	Yes	Higher quality
McNeill *et al*. 2022 [9]	Yes	Partial yes	No	Yes	N/A	Yes	N/A	Higher quality
Meernik *et al*. 2019 [39]	No	Partial yes	No	Partial yes	N/A	No	N/A	Lower quality
Notley *et al*. 2022 [11]	Yes	Yes	No	Yes	N/A	Yes	N/A	Higher quality
Novelli *et al*. 2022 [40]	No	Partial yes	No	Partial yes	N/A	Yes	N/A	Lower quality
Pepper and Brewer 2014 [41]	No	Partial yes	No	Partial yes	N/A	No	N/A	Lower quality
Riley *et al*. 2016 [42]	No	Partial yes	No	Yes	N/A	Yes	N/A	Lower quality
Sharma *et al*. 2021 [43]	Yes	Partial yes	No	No	N/A	No	N/A	Lower quality
Ward *et al*. 2020 [44]	No	Partial yes	No	Partial yes	N/A	No	N/A	Lower quality
Wilson *et al*. 2022 [45]	No	Partial yes	No	Partial yes	N/A	Yes	N/A	Lower quality
Yan *et al*. 2023 [46]	Partial yes	Partial yes	No	Yes	N/A	Yes	N/A	Higher quality
Yang *et al*. 2020 [47]	No	Partial yes	No	Partial yes	N/A	Yes	N/A	Lower quality
Yoong *et al*. 2021a [48]	Yes	Yes	No	Yes	Yes	No	No	Lower quality
Yoong *et al*. 2021b [49]	Yes	Partial yes	No	Yes	Yes	Yes	Yes	Higher quality

Abbreviations: AMSTAR‐2, A Measurement Tool to Assess Systematic Reviews‐2.

### Appeal and perceptions of vaping and motivation to use

Thirteen reviews covered outcomes related to the impact of flavours on appeal and perceptions of vaping and motivation to try or continuing vaping. Of these, eight reviews included overlapping studies. Of the 121 unique primary studies that contributed data on the impact of flavours on appeal and perceptions outcomes, 23 were included in more than one review. For information on study overlap, see Tables S1, S2 and S3. Information on specific flavours was limited. Most considered ‘non‐tobacco flavours’ as a category, rather than delving into specific flavours—where reviews referred to specific flavours, we note this below and in Table 3.

**TABLE 3:** Appeal.

Review ID	Flavour type	Population	Association direction^a^	Supporting information	Review quality
People with a history of combustible tobacco use					
Gades *et al*. 2022 [13]	Sweet; menthol/mint; general availability of (non‐tobacco) flavours	Adult current/former cigarette/e‐cigarette users	↑	‘Flavours, especially fruit, candy, and menthol/mint, increase the abuse potential and appeal of e‐cigarettes through increasing sweetness or coolness and decreasing bitterness and harshness of the product.’	Lower
	Tobacco flavours	People who smoke non‐menthol cigarettes and dual users	↑	‘Tobacco flavours, however, were appealing among non‐menthol cigarette smokers and dual users’	
Gentry *et al*. 2019 [30]	General availability of (non‐tobacco) flavours	Vulnerable groups* who smoke	↔	‘Views on…flavour were mixed’	Higher
Kowitt *et al*. 2017 [34]	General availability of (non‐tobacco) flavours	Adults who smoke tobacco	↑	‘In one US study, two e‐cigarette users (out of 50 total participants) noted that they felt that flavours played an instrumental (positive) role in cigarette smoking cessation, stating’ ‘If I do not like the flavour, I'm going to smoke a cigarette in a weird way, because it's not satisfying. It's like I'm a slave to nicotine, but if you find a flavour that you like, you are more inclined to be like, “This is sufficient. I do not want (a cigarette)”’	Lower
McNeill *et al*. 2022 [9]	Sweet flavours; menthol/mint flavours	Adults who smoke tobacco	↑	‘Mint and mango were rated as more satisfying than Virginia tobacco and creme’; ‘menthol flavoured e‐liquid was rated as more enjoyable than vanilla and tobacco flavoured e‐liquid’; ‘flavours appeared to independently affect satisfaction and subjective effects’	Higher
Population not selected for history of combustible tobacco use					
Kowitt *et al*. 2017 [34]	General availability of (non‐tobacco) flavours	Adolescents and young adults	↑	‘Two studies from the US also mentioned how flavours allowed e‐cigarette users to be more social, for instance, by serving as a conversation starter or by allowing people to share flavours in social settings.’	Lower
Meernik *et al*. 2019 [39]	General availability of (non‐tobacco) flavours	Young people; mixed tobacco use histories	↑	‘Among youth, flavours increase not only preferences for e‐cigarettes but they also increase e‐cigarette product appeal, willingness to use, susceptibility to use and initiation…’; ‘youth prefer non‐tobacco‐flavoured e‐cigarettes flavours—particularly sweet flavours such as fruit and candy…’	Lower
	General availability of (non‐tobacco, non‐menthol) flavours	Adults; mixed tobacco use histories	↑	‘e‐cigarette flavours increase product appeal and enjoyment.’; ‘non‐menthol flavours in e‐cigarettes increase appeal, enjoyment and the price users are willing to pay for the product’	
Notley *et al*. 2022 [11]	General availability of (non‐tobacco) flavours	Young people; mixed tobacco use histories	↑	‘There was a consistent theme of enjoyment with experimentation with different flavours, preferring to switch between flavours and try new flavours rather than sticking to one preferred flavour, suggesting the importance of variety and choice’; ‘There were different aspects to flavours that were attractive to young people that the qualitative data highlighted—enjoying the names, the descriptions, the labels and designs, as well as the actual taste’	Higher
Pepper and Brewer 2014 [41]	General availability of (non‐tobacco) flavours	Mixed age groups and tobacco use histories	↑	‘Users often mentioned taste and flavour. For example, more than 90% of users surveyed by Etter and Bullen liked the taste of ENDS.’; ‘A small number of users stopped using ENDS because they did not have the same flavour, but others preferred the flavour of ENDS to regular cigarettes.’	Lower
Sharma *et al*. 2021 [43]	General availability of (non‐tobacco) flavours	Adolescents; mixed tobacco use histories	↑	‘A total of 6 studies (24%; 2 quantitative and 4 qualitative) reported that adolescents considered the flavours and vapour to be appealing. […] Experimentation with and the use of E‐cigarettes were considered a fun social activity with friends where flavours […] could be trialed and discussed.’	Lower

Abbreviations: e‐cigarette, electronic cigarette; US, United States.

^a^ Direction of association between flavour class and appeal: ↑ = increased appeal; ↔ = no clear impact on appeal.

^b^ For example, experience of substance misuse substances, mental illness, homelessness or the criminal justice system.

Eight reviews contained some information relating to appeal (three higher quality; five lower quality) (Table 3). Of these, three reported that availability of non‐tobacco flavours increased the appeal of e‐cigarettes in people with a history of combustible tobacco use. The fourth review looking at this outcome reported mixed evidence. One review reported that among people who smoke non‐menthol tobacco, tobacco flavours were also appealing. In mixed populations, which also include people with and without a history of combustible tobacco use, all five relevant reviews reported non‐tobacco flavours being a particularly appealing aspect of e‐cigarette use. There was no clear difference in findings between higher and lower quality reviews.

Five reviews contained information on the impact of flavours on motivation to try or continue using e‐cigarettes (two higher quality; three lower quality) (Table 4). Two reviews reported that non‐tobacco flavours led to increased motivation in people with a history of combustible tobacco use, one showing increased motivation for continued use among pregnant women, and another showing increased motivation to start e‐cigarette use among young people. In mixed populations, including people with and without a history of combustible tobacco use, three reviews reported non‐tobacco flavours as a motivator to use e‐cigarettes, and one did not find evidence of difference in motivation among male adolescents when offered either an e‐cigarette explicitly described as flavoured or simply an e‐cigarette without mention of flavour. There was no clear difference in findings between higher and lower quality reviews.

**TABLE 4:** Motivation to try or continue using e‐cigarettes.

Review ID	Flavour type	Population	Association direction^a^	Supporting information	Review quality
People with a history of combustible tobacco use					
Campbell *et al*. 2020 [24]	General availability of (non‐tobacco) flavours	Pregnant women	↑	(Qualitative evidence) ‘Finding 18: perceived characteristics of e‐cigarettes … can influence uptake and continuous use of e‐cigarettes during pregnancy’ ‘They also found the selection of flavours particularly exciting and satisfactory.’	Higher
Kinouani *et al*. 2020 [33]	General availability of (non‐tobacco) flavours	Young adults who use or have tried e‐cigarettes	↑	One cross‐sectional study reported 24.6% (*n* = 2720) of current or former combustible tobacco users responded that flavours were a motivator for initiating.	Lower
Population not selected for history of combustible tobacco use					
Feirman *et al*. 2016 [29]	General availability of (non‐tobacco) flavours	Male adolescents; 57% with history of combustible tobacco use	↔	In one study participants were asked: ‘If one of your best friends were to offer you an e‐cigarette, would you try it?’ and ‘If one of your best friends were to offer you a flavoured e‐cigarette (chocolate, mint, apple, etc.), would you try it?’ ‘13.2% of participants answered that they were “probably” or “definitely” willing to try both types of e‐cigarettes. 3.9% of participants answered that they were “probably” or “definitely” willing to try the flavoured e‐cigarettes, but not the e‐cigarettes that were not specifically described as “flavoured.” 1.3% answered that they were “probably” or “definitely” willing to try the e‐cigarettes that were not specifically described as “flavoured,” but not the flavoured e‐cigarettes. The difference between the number of participants willing to try a flavoured e‐cigarette versus the number willing to try an e‐cigarette that was not described as “flavoured” did not significantly differ (*P* = 0.15).’	Lower
Kinouani *et al*. 2020 [33]	General availability of (non‐tobacco) flavours	Young adults who use or have tried e‐cigarettes	↑	Motivation for continued use: ‘A cross‐sectional study […] showed that reasons for using e‐cigarettes some days or daily amongst those aged 18–24 were, in decreasing order: smoking cessation or health; consideration for others; e‐cigarette convenience; curiosity; flavours; cost; and simulation of tobacco cigarettes. Using e‐cigarettes regularly was less associated with smoking cessation or health but more associated with flavours amongst adults aged 18–24.’	Lower
Kowitt *et al*. 2017 [34]	General availability of (non‐tobacco) flavours	Mixed age groups and tobacco use histories	↑	‘In three studies with young adults and adolescents from the US and Scotland, participants mentioned how flavours contributed to their initiation, experimentation and/or continued use of e‐cigarettes or other tobacco products’	Lower
Meernik *et al*. 2019 [39]	General availability of (non‐tobacco) flavours	Young people; mixed tobacco use histories	↑	‘[T]he availability of appealing flavours is associated with an increased willingness to try e‐cigarettes’	Lower
	General availability of (non‐tobacco, non‐menthol) flavours	Adults; mixed tobacco use histories	↑	‘[N]on‐menthol flavours in e‐cigarettes are a primary reason many adults use e‐cigarettes.’	

Abbreviation: e‐cigarette, electronic cigarette.

^a^ Direction of association between flavour class and motivation to use e‐cigarettes: ↑ = increased motivation; ↔ = no clear impact on motivation.

Five reviews contained information on the impact of flavours on perceptions of harm from e‐cigarette use (all lower quality) (Table 5). All investigated adolescents and young adults except Erku *et al*. 2020 [28], which focussed on healthcare professionals. In one review of young adult users of hookah, there were increased concerns about the potential of tobacco‐flavoured e‐cigarettes to lead to a ‘gateway effect’ to using combustible tobacco [34]. A review of healthcare professionals reported concerns over the potential impact of non‐tobacco flavours on respiratory health and reported healthcare professionals recommending restrictions of flavours [28]. In mixed populations, including people with and without a history of combustible tobacco use, two reviews reported flavoured e‐cigarettes being perceived as less harmful than tobacco‐flavoured e‐cigarettes. Among people with e‐cigarette use history but no history of combustible tobacco use, one review found that people described as non‐Hispanic Blacks and Hispanics perceived non‐tobacco flavoured e‐cigarettes as less harmful than tobacco‐flavoured e‐cigarettes compared with non‐Hispanic White/other participants [20].

**TABLE 5:** Perceptions of harm.

Review ID	Flavour type	Population	Association direction^a^	Supporting information	Review quality
People with a history of combustible tobacco use					
Kowitt *et al*. 2017 [34]	Tobacco flavours	Young adult users of hookah	↑	In one study there were concerns that tobacco flavoured e‐cigarettes may act as a gateway to other tobacco products, stating, ‘Non‐smokers could then be encouraged to smoke and try tobacco, like actual cigarettes and tobacco flavoured shisha’.	Lower
People with no history of combustible tobacco use					
Ahuja *et al*. 2023 [20]	General availability of (non‐tobacco) flavours	Adolescent current e‐cigarette users	Not applicable as comparing perceptions between groups	‘compared to non‐Hispanic White/other participants, both non‐Hispanic Blacks (OR = 1.32; 95% CI = 0.97–1.79) and Hispanics (OR = 1.09; 95% CI = 0.84–1.42) were more likely to perceive flavoured e‐cigarettes as ‘less harmful,’ than non‐flavoured e‐cigarettes’ ‘compared to very well off participants, those who lived “comfortably” and were just getting by/nearly poor/poor were more likely to perceive flavoured e‐cigarettes as “less harmful” (OR = 1.21; 95% CI = 0.84–1.75 and OR = 1.28; 95% CI = 0.81–2.02)’ (No overall results available)	Lower
	General availability of (non‐tobacco) flavours	Adolescent ever e‐cigarette users	Not applicable as comparing perceptions between groups	‘Compared to non‐Hispanic White/other participants, both non‐Hispanic Blacks (OR = 1.27; 95% CI = 0.93–1.73) and Hispanics (OR = 1.05; 95% CI = 0.82–1.34) were more likely to perceive flavoured e‐cigarettes as “less harmful,” (results not statistically significant). No significant association was reported between socio‐economic status and e‐cigarette harm and addiction perception.’ (No overall results available]	
Healthcare professionals					
Erku *et al*. 2020 [28]	General availability of (non‐tobacco) flavours	Healthcare professionals	↑	‘HCPs involved with care of thoracic surgery patients were concerned about the effect of ENDS aerosols and flavours on the respiratory system’. HCPs also recommended restricting flavours	Lower
Population not selected for history of combustible tobacco use					
Meernik *et al*. 2019 [39]	General availability of (non‐tobacco) flavours	Young people; mixed tobacco use histories	↓	‘Among youth […] flavours result in decreased e‐cigarette product harm perceptions’	Lower
Sharma *et al*. 2021 [43]	Sweet flavours	Adolescents; mixed tobacco use histories	↓	‘One quantitative study reported that cherry and candy flavours were perceived as less harmful than tobacco‐flavoured e‐cigarettes’	Lower

Abbreviation: e‐cigarette, electronic cigarette.

^a^ Direction of association between flavour class and perceived harms e‐cigarettes: ↑ = increased perceived harms; ↓ = decreased perceived harms.

### Harms from vaping

Twelve reviews investigated the potential harms from flavourings in e‐cigarettes (three higher quality; nine lower quality). We summarize these by flavour type/categorization in Table 6, and review‐specific supporting information is presented in Table S4. Of these, three included overlapping studies. Of the 70 unique primary studies that we identified contributing data on the impact of flavours on harms, four were included in more than one review. For information on study overlap, see Table S5. Most reviews found increased risk of harms when investigating specific flavours or flavour components. This was mostly in the form of cell damage in in vitro experiments where cells were exposed to flavoured e‐cigarette liquid, chemical analyses observing potentially toxic components or reports of adverse events such as throat irritation. No reviews reported evidence of any serious harms from e‐cigarette flavourings in human participants. There was no clear difference in findings between higher and lower quality reviews.

**TABLE 6:** Harms.

Flavour type	N reviews	Association direction^a^	Supporting information
Flavours in general	2 higher quality, 4 lower quality	↑ (4 reviews) ↔ (2 reviews)	One review of human experimental evidence found no difference in evidence of harms from e‐cigarettes when removing flavoured e‐cigarettes in a sensitivity analysis [35]. One review of human observational evidence found no adverse effects from flavours [11]. A review of qualitative evidence reported some people reporting unpleasant effects attributed to flavours such as nausea or throat irritation [34]. Two reviews of in vitro experimental evidence reported increased cariogenic bacteria and inflammation responses [23, 47]. A review of chemical analyses reported finding various toxicants such as formaldehyde and carbonyls [44].
Specific flavours			
Apple	1 lower quality	↑	One review of in vitro experiments reported DNA damage and reduced cell viability after exposure of oral pharyngeal cells to apple flavoured e‐cigarette liquid [45].
Banana	1 lower quality	↑	One review of in vitro experiments reported alterations in epithelial cell viability after exposure to banana flavoured e‐cigarette liquid [40].
Cherry	2 lower quality	↑ (1 review) ↔ (1 review)	One review of in vitro experiments reported DNA damage and reduced cell viability after exposure of oral pharyngeal cells to cherry flavoured e‐cigarette liquid [45]. One review of chemical analyses of e‐cigarette liquids reported high levels of benzaldehyde in the majority of cherry flavoured e‐cigarette liquids [44].
Chocolate	1 lower quality	↑	One review of in vitro experimental evidence reported that chocolate flavourings altered the salt and water balance at the airway surface, and lead to cell death, compromising the airway epithelium compared with other flavours [40].
Cinnamon	1 higher quality, 3 lower quality	↑	Three reviews of in vitro experiments and chemical analyses reported cinnamon flavour cytotoxic and associated it with suppressed bronchial ciliary function and temporarily suppresses bronchial epithelial cell ciliary motility by dysregulation of mitochondrial function [9, 40, 44]. One review of observational data associated long term use with mouth irritation [47]. Human experimental evidence form one review an increased rates of a bad reaction [9].
Citrus	2 lower quality	↑	One review of chemical analyses reported increased free radicals in aerosols generated from citrus e‐cigarette liquid [44]. A review of human observational evidence reported some e‐cigarette users describing negative throat symptoms associated with citrus e‐cigarette liquids [47].
Cola	1 lower quality	↑	One review of observational evidence reported some e‐cigarette users describing negative throat symptoms associated with cola flavoured e‐cigarettes [47].
Cotton candy	1 lower quality	↑	One review of in vitro experiments reported increased biofilm formation in cells exposed to cotton candy flavours compared to an unflavoured e‐liquid control [47].
Cucumber	1 lower quality	↑	One review of in vitro experimental evidence reported that cells exposed to cool cucumber flavour was reacted with higher mitochondrial stress, inflammatory cytokines, increased ROS and barrier dysfunction than other flavours [40].
Custard	1 lower quality	↑	One review of observational evidence reported some e‐cigarette users describing negative throat symptoms associated with custard flavoured e‐cigarettes [47].
Grape	1 lower quality	↑	One review of in vitro experimental evidence reported that grape flavour liquid, regardless of nicotine content adversely effected cell growth [47].
Mango	1 lower quality	↔	One review of chemical analyses observed cadalene in mango flavour e‐cigarette liquid [44].
Menthol/mint	5 lower quality	↑ (4 reviews) ↔ (1 review)	Three reviews of in vitro experiments reported various toxic effects from menthol flavour, including reduced cell viability, damaged DNA and induced mitochondrial dysfunction [40, 45, 47]. A review of chemical analyses reported higher rates of benzene and toluene than tobacco flavour [44]. A review of human experimental evidence found no significant differences in observations following a lab vaping session [42].
Pineapple	1 lower quality	↑	One review of in vitro experiments reported increased biofilm formation in cells exposed to pineapple flavours compared with an unflavoured e‐liquid control [47].
Tobacco	3 lower quality	↑	Two reviews of in vitro experimental evidence reported effects of tobacco flavouring on enamel colour and reduced cell viability, but there was disagreement on whether it resulted in DNA damage [45, 47]. One review of chemical analyses observed formaldehyde and acetaldehyde and free radicals [44].
Vanilla	1 lower quality	↑	One review of in vitro experimental evidence reported that vanilla flavourings altered the salt and water balance at the airway surface, and lead to cell death, compromising the airway epithelium compared with other flavours [40].
Types of flavours			
Buttery/creamy flavours	1 higher quality	↑	One review of in vitro and animal experimental evidence reported that buttery/creamy flavours have the potential to alter cellular responses but less than exposure to tobacco smoke [9].
Smokey flavours	1 lower quality	↑	One review of in vitro experiments reported increased biofilm formation in cells exposed to smokey flavours compared to an unflavoured e‐liquid control [47].
Sour flavours	1 lower quality	↑	One review of observational evidence reported some e‐cigarette users describing negative throat symptoms associated with sour flavoured e‐cigarettes [47].
Sweet/fruit flavours	1 higher quality, 1 lower quality	↑	One review of in vitro experimental evidence reported that fruit flavours were associated with DNA fragmentation and increased cariogenic potential [47]. A review of human experimental evidence reported higher concentrations of the biomarker for acrylonitrile [9].
Velvety flavours	1 lower quality	↑	One review of in vitro experiments reported increased biofilm formation in cells exposed to velvety flavours compared to an unflavoured e‐liquid control [47].

Abbreviation: e‐cigarette, electronic cigarette.

^a^ Direction of association between flavour and harms: ↑ = increased harm; ↔ = no clear impact on harms.

### Vaping behaviours

Fourteen reviews covered outcomes relating to the impact of e‐cigarette flavours on vaping behaviours, such as uptake or continued use of e‐cigarettes or reported on flavour choice within included cohorts. Of these, seven included overlapping studies. Of the 72 unique primary studies we identified that contributed data on the impact of flavours on vaping behaviours, seven were included in more than one review. For information on study overlap, see Table S6.

Four reviews provided information on the impact of flavours on e‐cigarette uptake (two higher quality; two lower quality) (Table 7). All described ‘flavours’ as a general category, rather than specifying flavour type or category. Two indicated that the availability of flavours may promote uptake of e‐cigarette use, whereas two reviews found mixed or inconclusive evidence. There was no clear difference in findings between higher and lower quality reviews.

**TABLE 7:** Vaping uptake.

Review ID	Evidence type	Association direction^a^	Supporting information	Review quality
‘Flavours’ as a general category				
Dautzenberg *et al*. 2023 [26]	Human observational	↑	‘Several studies in adolescents or young adults suggest that flavours promote the uptake of e‐cigarettes because they appear less hazardous and more appealing, especially when there are numerous flavours to choose from. The authors conclude that this may increase nicotine dependence and could favour the transition to tobacco use.’	Lower
Han *et al*. 2022 [31]	Human observational	↔	‘Four studies on the flavours in e‐cigarettes showed that flavour was a risk factor for e‐cigarette use, and three studies indicated no association’	Lower
Notley *et al*. 2022 [11]	Human observational	↑	‘There were six longitudinal studies following the same cohort overtime able to robustly assess a possible causal association between first use of a flavoured e‐liquid and subsequent uptake of regular e‐cigarette use. Overall, these six studies suggested a positive association between the first use of a flavoured e‐liquid product and continued vaping.’	Higher
Yoong *et al*. 2021b [49]	Human observational	↔	‘One study reported no difference in uptake of ENDS/ENNDS use at follow up between flavoured vs unflavoured e‐cigarette use at baseline (RR: 0.24 (95% CI = 0.05, 1.0) when controlling for sex, age, state, school type, migration background, parent's qualifications, socio‐economic status (SES), multiple personality traits and consumption of five substances’	Higher

Abbreviations: e‐cigarette, electronic cigarette; SES, socio‐economic status.

^a^ Direction of association between flavour and harms: ↑ = increased uptake; ↔ = no clear impact on uptake.

Ten reviews provided information on e‐cigarette flavour selection and preference (five higher quality; five lower quality) (Table S7). Among these reviews, fruit and sweet flavours were consistently popular. Tobacco flavours were often, although not always, more popular than non‐tobacco flavours among current/former combustible tobacco users and older users. Some evidence reported mint and menthol flavoured e‐cigarettes being more popular than other flavours among people who had smoked menthol flavoured cigarettes. Across several reviews there was evidence that flavour preferences varied based on a range of factors including age, combustible tobacco use history, country and even device used.

Lindson *et al*. [1, 37, 38] (higher quality) conducted a subgroup analysis testing the effect of flavours on long‐term study product use in trials comparing e‐cigarettes with nicotine replacement therapy for smoking cessation. This subgroup analysis divided those studies where participants were randomized to receive tobacco flavoured e‐cigarettes and those where participants were randomized to receive e‐cigarettes with a choice of tobacco, menthol or sweet flavours. This subgroup analysis found evidence of heterogeneity between groups (I^2^ = 65.2%), with the ‘tobacco’ subgroup reporting more participants using e‐cigarettes than nicotine replacement therapy (NRT) at long‐term follow‐up. The ‘choice’ subgroup had a substantially smaller point estimate favouring higher long‐term e‐cigarette use, with CI encompassing the potential for higher long‐term NRT use as well as no difference.

Dyson *et al*. [27] (lower quality) reported that e‐cigarette users claimed that not liking the flavour of their e‐cigarette was a facilitator to quitting e‐cigarette use. This appeared to be the case in middle schoolers more than in high school or college students. They also reported that ‘study participants had a range of suggestions relating e‐cigarette cessation support including […] switching to an alternative e‐cigarette flavour, for example, using tobacco flavour first to ease the process from cigarette to e‐cigarette and then an alternative flavour to progress to e‐cigarette cessation’ [27]. Meernik *et al*. [39] (lower quality) reported that users with a preference for non‐tobacco flavours appeared to use e‐cigarettes more frequently.

Banks *et al*. [21] (lower quality) reported that among combustible tobacco users there was insufficient evidence to conclude whether e‐cigarette abuse liability is influenced by flavour.

### Use of other forms of tobacco/nicotine

Six reviews reported outcomes related to combustible tobacco use, such as combustible tobacco smoking cessation or uptake. Of these, five included overlapping studies. Of the 54 unique primary studies that contributed data on the impact of flavours on combustible tobacco use, five were included in more than one review. For information on study overlap, see Tables S8 and S9. No reviews reported on the impact of e‐cigarette flavours on use of tobacco or nicotine products other than combustible tobacco.

Six reviews provided information on cessation or reduction of combustible tobacco smoking (four higher quality; two lower quality) (Table 8). One review reported that based on observational evidence, young people who used one or multiple non‐tobacco non‐menthol flavours were more likely to have reduced or stopped cigarette use during the past year than e‐cigarette non‐users. However, there was no comparison with unflavoured or other e‐cigarette use. Of the remaining five reviews, including all of the higher‐quality reviews and including those comparing non‐tobacco flavoured e‐cigarette use with tobacco‐flavoured e‐cigarette use, one high‐quality review found association between flavours and increased smoking cessation [49], and the remaining four reviews did not.

**TABLE 8:** Combustible tobacco cessation/reduction.

Review ID	Flavour type	Population	Association direction^a^	Supporting information	Review quality
Ciapponi *et al*. 2021 [25]	Non‐tobacco non‐menthol flavours	Young adults who use combustible tobacco	Not applicable as comparing flavoured e‐cigarettes with non‐users	‘Chet *et al*. reported that e‐cigarette users with one (adjusted OR = 2.5; *P* < 0.001) or multiple tobacco‐free/menthol‐free flavours (adjusted OR = 3.0; *P* < 0.001) were more likely to have reduced or stopped cigarette use during the past year compared with non‐users of e‐cigarettes’	Lower
Liber *et al*. 2023 [36]	Non‐tobacco flavours	Combustible tobacco users	↔	‘Using the GRADE approach, we found that there were low levels of certainty that using flavoured ENDS compared to non‐flavoured or tobacco‐flavoured ENDS did not increase the likelihood of intending to quit or making a quit attempt. We found a very low level of certainty that persons using nontobacco ENDS did not have a higher likelihood of quitting success compared to those using tobacco/unflavoured ENDS. We reached similar findings for non‐menthol and nontobacco flavours compared to tobacco and menthol‐flavoured ENDS’	Higher
Lindson *et al*. 2024 [1]	All	Combustible tobacco users	↔	‘One study […] randomised participants to […] EC with tobacco flavour pods and EC with a choice of sweet, tobacco or menthol pods. At 12 month follow‐up, 51/285 (17.9%) participants in the tobacco pod study arm and 40/281 (14.2%) participants in the flavour choice arm reported 30‐day point prevalence smoking abstinence (RR = 1.26; 95% CI = 0.86–1.84).’ ‘Subgrouping by our specified flavour categories did not show evidence of effect moderation.’ ‘The flavour most used by cigarette abstainers varied between studies (see figure 4). In one UK study, the majority of people who were abstinent from smoking were using sweet flavours, with smaller, very similar numbers using menthol/mint and tobacco flavours. In a US study, the participants who chose sweet (mango) flavour at baseline were most likely to be abstinent from cigarettes at 6‐month follow‐up [54% quit versus 38% menthol/mint and 37% tobacco flavours; see figure 3(a)]; most abstainers were using sweet/fruit, or menthol/mint flavours at follow‐up (figure 4). A second US study reported that 30‐day point prevalence quit rates at 12‐month follow‐up were similar across primary flavours chosen in the relevant study arm (using complete case analysis): tobacco 4/25 (16%); menthol/mint 5/28 (18%); sweet 7/36 (19%); and a UK study, Begh 2021, found that of the four participants who quit, two were using sweet flavours, one tobacco flavour and one menthol/mint flavour. In an Italian study, sweet flavours appeared to be used the least by cigarette abstainers at follow‐up, with tobacco the most popular flavour. Three of the studies also provided the number of people using each EC flavour who were not abstinent ≥6 month follow‐up (figure 4). Among the non‐abstinent group, flavour use was evenly matched in one study, with nine participants using tobacco; 10 using mint; and nine using sweet flavours. In the others, tobacco flavour seemed to be less popular at follow‐up, with mint/menthol the most popular in one, and sweet the most popular in the other. As in our subgroup analyses, it is important to treat the data on abstinence and flavours with caution as the groupings of participants and the events occurring within them were relatively small for all studies, and are observational in nature.’	Higher
Meernik *et al*. 2019 [39]	Non‐tobacco and non‐menthol flavours	Young people who use combustible tobacco	↔	‘In regards to smoking cessation, one national probability sample of 21 491 youth in the USA found that among current smokers, students who reported using flavoured e‐cigarettes were less likely to quit [combustible] tobacco use compared with those who reported not using e‐cigarettes or with those who had used non‐flavoured e‐cigarettes’ ‘Evidence on whether non‐menthol‐flavoured e‐cigarettes promote or disrupt cessation among adult smokers remains unclear.’ (Mixed results from 7 studies) Youth and adults – ‘one study found menthol and coffee users were more likely to quit’	Lower
Notley *et al*. 2022 [11]	Any flavour	Young people who use combustible tobacco	↔	A study looking at the effect of flavours on smoking cessation was inconclusive and ‘the role of flavours in tobacco smoking uptake or cessation is unclear’	Higher
Yoong *et al*. 2021b [49]	Any flavour	Adults who use combustible tobacco	↑	Includes a study that associated vaping nontobacco flavours with increased adult smoking cessation (AOR in adults, 2.28; 95% CI = 1.04–5.01; *P* = 0.04).	Higher

Abbreviations: AOR, adjusted OR; EC, e‐cigarettes; e‐cigarette, electronic cigarette; UK, United Kingdom; US, United States; USA, United States of America.

^a^ Direction of association between flavour and combustible tobacco cessation/reduction: ↔ = no clear impact.

Two higher quality reviews provided information on the impact of e‐cigarette flavours on combustible tobacco uptake. Neither found clear evidence of an association (Table 9).

**TABLE 9:** Combustible tobacco initiation.

Review ID	Flavour type	Population	Association direction^a^	Supporting information	Review quality
Notley *et al*. 2022 [11]	Any flavour	Young people	↔	In the one longitudinal study, Friedman, reporting 48‐month follow‐up data, found no association with flavoured e‐liquid use and subsequent tobacco smoking initiation: ‘For both youths and emerging adults, the association of flavoured e‐cigarette use and smoking initiation was not significantly different from that for unflavoured e‐cigarette use (AOR for youth, 0.66; 95% CI = 0.16–2.76; *P* = 0.56; AOR for emerging adults, 3.15; 95% CI = 0.14–71.78; *P* = 0.46).’	Higher
Yoong *et al*. 2021b [49]	Any flavour	Young people who use combustible tobacco	↔	Includes a study that concludes ‘Vaping nontobacco flavours was no more associated with youth smoking initiation than vaping tobacco‐flavours (AOR in youth, 0.66; 95% CI = 0.16–2.76; *P* = 0.56)’	Higher

Abbreviations: AOR, adjusted OR; e‐cigarette, electronic cigarette.

^a^ Direction of association between flavour and combustible tobacco cessation initiation: ↔ = no clear impact.

### Impacts of flavour bans

Yan et al. [46] reported on the impacts of legislation affecting e‐cigarette flavours. Based on evidence from three primary studies, they found that flavour restrictions that prohibited all flavours other than tobacco and menthol, or restricted flavours to adult‐only stores, decreased flavoured e‐cigarette availability and resulted in decreased flavoured e‐cigarette sales and increased sales of unrestricted e‐cigarette products. They also resulted in more negative tweets about and fewer on‐line searches for e‐cigarette products.

## DISCUSSION

This overview shows that e‐cigarette flavours have a potential impact on a range of outcomes, although a general paucity of evidence precludes firm conclusions. Non‐tobacco e‐cigarette flavours may increase the appeal of e‐cigarettes and motivation to start or continue using e‐cigarettes and may decrease perceptions of harm. Flavour constituents may lead to increased harms, although evidence was very limited. Evidence was inconclusive on whether non‐tobacco flavours could influence the uptake/cessation of e‐cigarettes and combustible tobacco use. Given the sparsity of evidence, and that no reviews compared adult with younger populations, no firm conclusions or trends could be identified on differences in the evidence between these groups. Further research is needed to draw confident conclusions for all of these outcomes. High‐quality evidence from RCTs and well‐designed observational studies, with outcomes broken down by flavours, would be greatly beneficial.

Despite a proliferation of publications and systematic reviews relating to e‐cigarettes and their flavours, the evidence base is very limited. Reviews were mostly of lower quality—often because of limitations with pre‐registered protocols and risk of bias assessments. Evidence on flavours from reviews often came from very few studies, and much of the primary evidence reviews synthesized comprised study designs that are less likely to provide reliable insight into the real‐world impact of e‐cigarette flavours in people using them. In particular, the majority of evidence for potential harms from flavours came from chemical analyses of e‐cigarette liquids and in vitro experiments that exposed cells to the liquids in a lab setting. Where reviews synthesized more reliable forms of evidence regarding impacts on human health, such as trials or well‐designed observational evidence, the quality of evidence was limited by the paucity of primary evidence reporting desired outcomes broken down by flavours.

This overview has several potential limitations. As this is an overview of reviews, the timeline of the evidence presented here is limited to the search dates of the included reviews. As the most recent search date of any of the included reviews is February 2024, this overview potentially misses the most up to date evidence. The potential for overlap in primary evidence included in reviews runs the risk of overstating findings. We only found minimal overlap between included reviews, and given that we refrain from any firm conclusions based on this evidence, this only presents a minor limitation. Further, as an overview of reviews, this research inherits the limitations of the included reviews and their syntheses of the primary evidence. As most of the reviews were of a lower quality, this has the potential to bias the findings of this overview. Further, we extracted data from review write‐ups in good faith, without looking further into the primary evidence they synthesized. As such, the potential for human error is magnified, and where flavours were not the primary focus of an included review (as was the case for 25 reviews), we are likely missing insights from the primary literature.

In conclusion, the extant literature on flavours is limited, but does suggest they are an important characteristic, which may potentially impact a range of outcomes critical to their public health impact, including: e‐cigarette use; use of combustible tobacco; safety profile; appeal; and perceptions of harm. Further primary literature and systematic reviews are needed in this space, with a particular need for clear specification of flavour types and categories.

## AUTHOR CONTRIBUTIONS

**Jonathan Livingstone‐Banks:** Conceptualization (equal); data curation (lead); formal analysis (lead); funding acquisition (equal); investigation (equal); methodology (equal); project administration (lead); visualization (lead); writing—original draft (lead); writing—review and editing (lead). **Nargiz Travis:** Data curation (equal); investigation (equal); writing—review and editing (equal). **Monserrat Conde:** Data curation (equal); investigation (equal); writing—review and editing (equal). **Yixian (Crystal) Chen:** Data curation (equal); investigation (equal); writing—review and editing (equal). **Padmo Zi:** Data curation (equal); investigation (equal); writing—review and editing (equal). **Holly Jarman:** Data curation (equal); investigation (equal); writing—review and editing (equal). **Nicola Lindson:** Conceptualization (equal); data curation (equal); formal analysis (equal); funding acquisition (equal); investigation (equal); methodology (equal); visualization (equal); writing—original draft (equal); writing—review and editing (equal). **Jamie Hartmann‐Boyce:** Conceptualization (equal); data curation (equal); formal analysis (equal); funding acquisition (equal); investigation (equal); methodology (equal); visualization (equal); writing—original draft (equal); writing—review and editing (equal).

## DECLARATION OF INTERESTS

J.L.B., N.L. and J.H.B. are authors of one of the included reviews. J.H.B. reports research consultancy funding from the Truth Initiative. None of the other authors have interests to declare.

Research reported in this publication was supported by the National Cancer Institute of the National Institutes of Health (NIH) and US Food and Drug Administration (FDA) Center for Tobacco Products under award number 2U54CA229974. The content is solely the responsibility of the authors and does not necessarily represent the official views of the NIH or the FDA.

## Supporting information


**Table S1:** Appeal overlap.
**Table S2:** Motivation to try or continue using e‐cigarettes overlap.
**Table S3:** Perceptions of harm overlap.
**Table S4:** Harms results.
**Table S5:** Harms overlap.
**Table S6:** Vaping uptake overlap.
**Table S7:** Preference, use and/or selection of specific flavors or flavor groups.
**Table S8:** Combustible tobacco cessation/reduction.
**Table S9:** Combustible tobacco initiation.
**Table S10:** Excluded studies.

## Data Availability

The data that support the findings of this study are from published systematic reviews. These data were derived from the published reports, which are publicly available.
