# Supplementary material for: The impacts of e‐cigarette flavours: An overview of systematic reviews
Source: Addiction. 2025 Feb 25;120(7):1327–44. doi: 10.1111/add.70017 (PMC12128567; doi:10.1111/add.70017)
Supplement: Supplementary file 1 — Table S1: Appeal overlap. Table S2: Motivation to try or continue using e‐cigarettes overlap. Table S3: Perceptions of harm overlap. Table S4: Harms results. Table S5: Harms overlap. Table S6: Vaping uptake overlap. Table S7: Preference, use and/or selection of specific flavors or flavor groups. Table S8: Combustible tobacco cessation/reduction. Table S9: Combustible tobacco initiation. Table S10: Excluded studies. [file ADD-120-1327-s001.docx]

# Supplementary information

### Supplementary table 1: Appeal overlap

|  | **Gades 2022** *(32 studies)* | **Gentry 2019** *(3 studies)* | **Kowitt 2017** *(1 study)* | **McNeill 2022** *(2 studies)* | **Meernik 2019** *(11 studies)* | **Notley 2022** *(46 studies)* | **Pepper 2014** *(12 studies)* | **Sharma 2021** *(7 studies)* |
| --- | --- | --- | --- | --- | --- | --- | --- | --- |
| **Gades 2022** *(32 studies)* |  | 0 | 0 | 0 | 3 | 7 | 0 | 0 |
| **Gentry 2019** *(3 studies)* | 0 |  | 0 | 0 | 0 | 0 | 0 | 0 |
| **Kowitt 2017** *(1 study)* | 0 | 0 |  | 0 | 0 | 0 | 0 | 0 |
| **McNeill 2022** *(2 studies)* | 0 | 0 | 0 |  | 0 | 0 | 0 | 0 |
| **Meernik 2019** *(11 studies)* | 3 | 0 | 0 | 0 |  | 3 | 1 | 0 |
| **Notley 2022** *(46 studies)* | 7 | 0 | 0 | 0 | 3 |  | 0 | 5 |
| **Pepper 2014** *(12 studies)* | 0 | 0 | 0 | 0 | 1 | 0 |  | 0 |
| **Sharma 2021** *(7 studies)* | 0 | 0 | 0 | 0 | 0 | 5 | 0 |  |

### Supplementary table 2: Motivation to try or continue using e-cigarettes overlap

|  | **Campbell 2020** *(2 studies)* | **Feirman 2016** *(1 study)* | **Kinouani 2020** *(2 studies)* | **Kowitt 2017** *(3 studies)* | **Meernik 2019** *(12 studies)* |
| --- | --- | --- | --- | --- | --- |
| **Campbell 2020** *(2 studies)* |  | 0 | 0 | 0 | 0 |
| **Feirman 2016** *(1 study)* | 0 |  | 0 | 0 | 0 |
| **Kinouani 2020** *(2 studies)* | 0 | 0 |  | 0 | 1 |
| **Kowitt 2017** *(3 studies)* | 0 | 0 | 0 |  | 0 |
| **Meernik 2019** *(12 studies)* | 0 | 0 | 1 | 0 |  |

### Supplementary table 3: Perceptions of harm overlap

|  | **Ahuja 2023** *(1 study)* | **Erku 2020** *(3 studies)* | **Kowitt 2017** *(1 study)* | **Meernik 2019** *(5 studies)* | **Sharma 2021** *(1 study)* |
| --- | --- | --- | --- | --- | --- |
| **Ahuja 2023** *(1 study)* |  | 0 | 0 | 1 | 0 |
| **Erku 2020** *(3 studies)* | 0 |  | 0 | 0 | 0 |
| **Kowitt 2017** *(1 study)* | 0 | 0 |  | 0 | 0 |
| **Meernik 2019** *(5 studies)* | 1 | 0 | 0 |  | 1 |
| **Sharma 2021** *(1 study)* | 0 | 0 | 0 | 1 |  |

### Supplementary table 4: Harms results

| **Review ID** | **Evidence type** | **Association direction** | **Supporting information** | **Review quality** |
| --- | --- | --- | --- | --- |
| ***“Flavours” as a general category*** | | | | |
| Kowitt 2017 | Qualitative | ↑ | "A few participants described unpleasant effects of flavoring (e.g., nausea, throat irritation, burning sensation)." [one study] | Lower |
| Larue 2017 | Human experimental | ↔ | "This review found that acute exposure to e-cigs did affect several cardiovascular and respiratory measures. Compared to combustible cigarettes, the use of e-cigs with nicotine was associated with a similar significant increase in HR, BP and arterial stiffness (AIx75). There was also a significant decrease in FeNO although less pronounced than the one following combustible cigarettes. In addition, there was a trend for a decrease in FEV1 in response to both e-cigs with and without nicotine."  "Sensitivity analysis focusing on healthy volunteers and standard ecigs (i.e., removing populations who were defined as having a disease and analyses conducted on flavored e-liquid) didn’t impact the physiological changes previously observed." | Lower |
| Camoni 2023 | In vitro experimental | ↑ | "Articles regarding the effects of ENDS on cariogenic bacteria [three studies] concluded that flavored liquids from e-cigarette are detrimental to oral health, and an effect on enamel similar to that of gelatinous sweets or acidic drinks has been speculated"  "It has been found that flavored e-liquids are often sweeter and stickier than unflavored ones, promoting bacterial adhesion and reducing normal commensal flora, with dysbiosis of the oral microbiome [three studies]" | Higher |
| Notley 2022 | Human observational | ↔ | "We found no included reports of adverse effects of flavours." | Higher |
| Ward 2020 | Chemical analyses | ↑ | “Aerosol toxicants are also associated with e-liquid flavoring agents existing as primary ingredients or as products of thermal degradation".  "Kim et al. [51] observed that carbonyls were present in aerosol associated with major flavoring groups but not in un-aerosolized liquid... Klager et al. [97] observed median formaldehyde concentrations of 626 μg/m3 in aerosol produced with 24 e-liquid flavors”  “Qu et al. [74] observed that the carbonyl compounds emission factors increased linearly 1.0- to 92-fold when flavor content of the e-liquid increased from 5% to 50% and concluded that most carbonyls in e-liquids were from flavorings....”  “Vreeke et al. [35] observed that e-liquid containing 10% flavoring compound was associated with increased carbonyls in aerosol compared with e-liquids containing only VG:PG [propylene glycol (PG), vegetable glycerin (VG)]. Duell et al. [39] observed that the concentrations of aldehydes (propionaldehyde, acetaldehyde, glycolaldehyde, and acrolein) increased as sucralose [used in flavourings] concentration in the e-liquid increased....Reilly et al. [84] observed no differences in formaldehyde and acetone across the aerosols of 4 different JUUL flavors"  "..Vreeke et al. [35] reported that the addition of triacetin, a common flavor additive, increased levels of formaldehyde hemiacetals. Duell et al. [39] observed that formaldehyde hemiacetals increased with increasing concentrations of sucralose added to the e-liquid"  "Beauval et al. [60] evaluated 6 e-liquid refills...The concentrations of metals varied minimally with the presence or absence of flavoring and nicotine. In contrast, Mikheev et al. [86] observed that that As, Cr, Ni, Cu, Sb, Sn, and Zn levels in ENDS aerosol varied widely across nicotine- and non-nicotine-containing flavors."  Bitzer et al. [58] “analyzed the free radicals generated from 49 commercially available e-liquids and observed that nearly 43% of the flavors resulted in significant increases in radical production compared with the base VG:PG (40:60) mixture, but the amount varied greatly among the flavors; the flavorings dipentene, ethyl maltol, citral, linalool, and piperonal promoted radical formation in a concentration-dependent manner, whereas ethyl vanillin inhibited radical formation. Overall, ROS and free radicals in ENDS aerosol vary by e-liquid composition, but the precise relationship has yet to be determined." | Lower |
| Yang 2020 | In vitro experimental | ↑ | “Exposure to e-cigarette aerosols with flavorings was associated with increases in pro-inflammatory cytokines and inflammation in periodontal ligament fibroblasts, human gingival epithelium progenitors, and a human epigingival tissue model (Sundar et al. 2016)."  According to another study, “Exposure to flavored e-cigarette aerosols may potentiate cariogenic bacteria […] and was also associated with a 27% decrease in enamel hardness compared to unflavored control aerosols." | Lower |
| ***Comparing multiple flavours*** | | | | |
| Lindson 2024 | Human experimental | ↔ | “One study (Edmiston 2022, n = 300, high risk of bias, vaping/tobacco industry funding) randomized participants to different flavours (tobacco versus menthol) and provided SAE data in a way that could have been used to compute risk ratios, although no SAEs occurred in either arm (Analysis 8.1; 0/150 versus 0/150). NNAL and FEV1/FVC were lower in the tobacco flavour group but CIs were wide and included no difference (Analysis 8.2 [MD -26.10, 95% CI -66.73 to 14.53]; Analysis 8.4 [MD -0.46, 95%CI -1.67 to 0.75]). There was no evidence of a difference in FEV1 (Analysis 8.3 [MD-0.67, 95% CI -2.34 to 1.00). No other outcomes from this paper were eligible for inclusion in our review.  Morris 2022, a randomized cross-over trial, tested the effect of 10 different flavours (as well as nicotine strengths and salt versus free-base nicotine). Only their data on AE and SAE were eligible for inclusion in our review, but analyses were not reported by flavour (Supplemental Table 1; Supplemental Table 2).  White 2021 also contributed data to this comparison, with conditions being tobacco flavours only, or tobacco, fruit, dessert and mint flavours. No significant main effects or interactions were found for flavours on the outcomes relevant to this review, namely CO and CEMA, and no difference was discernable in the occurrence of AEs. However, as noted above, the study terminated early and hence was underpowered to detect differences.” | Higher |
| McNeill 2022 | Human observational | ↑ | "When asked “What flavour were you using at that time [of reporting perceived flavour associated adverse reactions]?” participants identified tobacco or menthol (n= 6), cinnamon (n= 5), fruit (n= 5), beverage (n= 2), other/not sure (n= 3), and high propylene glycol or vegetable glycerine (n= 5)" | Higher |
| ***Tobacco flavours*** | | | | |
| Ward 2020 | Chemical analyses | ↑ | One study “observed formaldehyde and acetaldehyde close to 1 ng/mL of emission with no substantial differences between flavorings, with the exception of acrolein concentrations from aerosol produced with “blond tobacco” flavored e-liquid.” Another study “observed that acrolein was present in higher concentrations in tobacco-flavored than in fruit-flavored e-liquid.”  Another study “analyzed aerosol from JUUL and observed that free radicals were 2.95 ± 0.81 nmol/g for tobacco flavor at a 70:30 VG:PG ratio [propylene glycol (PG), vegetable glycerin (VG)]; the concentration was higher (3.01 ± 0.28 nmol/g) when the eliquid composition was 70:30 VG:PG without flavoring.”  "Farsalinos et al. [91] observed that no TSNAs were above the level of detection for aerosols generated from 3 tobacco-flavored e-liquids aerosolized by a second-generation ENDS"  "Lee et al. [105] detected more carbonyls in ENDS aerosol generated from tobacco flavors than from menthol". | Lower |
| Wilson 2022 | In vitro experimental | ↑ | "Ji et al. examined normal human oral keratinocytes (NHOK’s) against e-cigarette vapor with and without nicotine and determined tobacco […] ecigarette vapor with and without nicotine reduced cell viability significantly (p < 0.05 […])."  "Sundar et al. concluded that tobacco […] e-cigarette vapor with nicotine-induced DNA damage in HPdLF’s (p < 0.05 […]).5" | Lower |
| Yang 2020 | In vitro experimental | ↑ | "...(Pintado-Palomino et al. 2019) assessed the impact of different e-liquid flavors (neutral, menthol, and tobacco) and nicotine concentrations on enamel color and found alterations in luminosity, particularly when enamel was exposed to e-liquids containing menthol and tobacco flavors."  Welz et al. (2016) demonstrated that fruit flavored e-liquids were associated with DNA fragmentation, whereas the tobacco flavored product was not | Lower |
| ***Cinnamon flavours*** | | | | |
| Novelli 2022 | In vitro experimental | ↑ | An article associated the flavour cinnamaldehyde with suppressed bronchial ciliary function - "The flavor additive cinnamaldehyde temporarily suppresses bronchial epithelial cell ciliary motility by dysregulation of mitochondrial function" | Lower |
| Ward 2020 | Chemical analyses | ↑ | "Behar et al. [103] found cinnamaldehyde [Cinnamaldehyde has been observed to be cytotoxic] in 20 (51%) of 39 refill fluids...Soussy et al. [104] reported that emissions of 5-hydroxymethylfurfural and furfural were correlated with device power and sweetener concentration in the e-liquid." | Lower |
| Yang 2020 | Human survey | ↑ | Sinharoy et al. (2018) found that in long-term e-cigarette users, there was an association between cinnamon flavor and mouth irritation." | Lower |
| McNeill 2022 | In vitro experimental and human experimental | ↑ | “Findings from the 13 cell and 9 animal studies suggest there is limited evidence that some flavourings in vaping products, particularly cinnamaldehyde and buttery/creamy flavours, have the potential to alter cellular responses but less than exposure to tobacco smoke. Exposure to unflavoured PG/VG base liquids appeared to have little or no effect.”  “In addition, 23.7% and 31.8% reported having ever used any e-liquid that contained either popcorn flavour/diacetyl or cinnamon flavour/cinnamaldehyde or 2-methoxicinnamaldehyde, respectively. Participants who had ever used these flavours (total n= 154) were more likely than nonusers to report having had a bad reaction (13.0% vs. 2.7%; p < 0.0001).” | Higher |
| ***Menthol/mint flavours*** | | | | |
| Novelli 2022 | In vitro experimental | ↑ | “Due to recent FDA regulations banning all flavor pods besides menthol and tobacco, one article examined these two flavors specifically and found the menthol flavor to induce significantly more mitochondrial dysfunction in lung epithelial cells" | Lower |
| Riley 2016 | Human experimental | ↔ | "In the crossover study, 20 participants (9 women) were randomized to receive ECs containing 18-mg nicotine/mL e-liquid in either menthol or nonmenthol tobacco flavor. Participants used ECs for 7–10 days in lieu of conventional cigarettes, returned to the laboratory for a monitoring session (5 min of EC use with heart rate and BP recorded) and then repeated the procedure with the alternative flavor. No significant increases to heart rate or BP were recorded in the 30 min of observation following either laboratory session, with mean heart rate ranging from ~66 to 69 bpm, mean systolic BP ranging from 120 to 125 mmHg and mean diastolic BP remaining ~ 75 mmHg throughout the observation periods." | Lower |
| Ward 2020 | Chemical analyses | ↑ | One study “observed that menthol flavor generated 330% more benzene and 120% more toluene than in tobacco flavor.”  One study “generated aerosols from 3 different e-liquids generated by a tank system and estimated freeradical production to be 10.3, 4.0 and 2.5 × 1013 radicals per puff of menthol, citrus, and tobacco flavors, respectively” | Lower |
| Wilson 2022 | In vitro experimental | ↑ | “Ji et al. examined normal human oral keratinocytes (NHOK’s) against e-cigarette vapor with and without nicotine and determined […] menthol ecigarette vapor with and without nicotine reduced cell viability significantly ([…]p < 0.05, respectively)."  "Sundar et al. concluded that […] menthol e-cigarette vapor with nicotine-induced DNA damage in HPdLF’s ([…]p < 0.01, respectively).” And "Increases in IL−8 and prostaglandins, was greatest for mint flavor e-cig vapor"  Willershausen et al. (2014) - Menthol flavored e-cigarette liquid has a toxic effect in human periodontal fibroblasts  Inkielewicz-Stepniak et al. (2016) - "Biological inhibitory effect on HGF−1 for mint flavored e-cigarette liquid" | Lower |
| Yang 2020 | In vitro experimental | ↑ | One study “found that the frequency of mouth irritation increased with menthol concentration, but not nicotine concentration.  “throat irritation or harshness appeared to be mediated by menthol, with a stronger symptomatology at low nicotine concentrations, and weaker symptomatology at higher nicotine concentrations, suggesting that menthol may mask or reduce the perception of airway irritation and harshness caused by high levels of nicotine (Rosbrook and Green 2016).  "...(Pintado-Palomino et al. 2019) assessed the impact of different e-liquid flavors (neutral, menthol, and tobacco) and nicotine concentrations on enamel color and found alterations in luminosity, particularly when enamel was exposed to e-liquids containing menthol and tobacco flavors."  “Willershausen et al.’s (2014) results of reduced cell proliferation and detection of ATP were a result of exposure to menthol flavored liquid.” | Lower |
| ***Sweet/fruit flavours in general*** | | | | |
| Yang 2020 | In vitro experimental | ↑ | "Kim et al. (2018) suggested that a combination of viscosity of e-liquids and sweet flavors may increase the cariogenic potential of e-cigarettes.”  “Welz et al. (2016) demonstrated that fruit flavored e-liquids were associated with DNA fragmentation, whereas the tobacco flavored product was not.”  "An in vitro study that analyzed the cariogenic potential for various flavored e-cigarette liquids tested five types of flavors (hexyl acetate-apple/plum, ethyl butyrate-pineapple, sucralose-sugar substitute, triacetin- “velvety” or “smoky” flavor, and ethyl maltol-cotton candy). Four out of five flavors (sucralose, ethyl butyrate, triacetin, hexyl acetate) significantly increased biofilm formation compared to an unflavored e-liquid control | Lower |
| McNeill 2022 | Human experimental | ↑ | “Users of fruit-only flavoured vaping products had significantly higher concentrations of the biomarker for acrylonitrile (CYMA) compared to users of a single other flavour in one study.” | Higher |
| ***Chocolate flavours*** | | | | |
| Novelli 2022 | In vitro experimental | ↑ | One study found chocolate (2,5-dimethypyrazine) flavorings altered CFTR ion conductance compared with other flavours - "Chocolate (2,5-dimethypyrazine) […] flavoring affect CFTR activation (altering the salt and water balance at the airway surface) and lead to cell death, compromising the airway epithelium." | Lower |
| ***Vanilla flavours*** | | | | |
| Novelli 2022 | In vitro experimental | ↑ | One study found vanillin flavorings altered CFTR ion conductance compared with other flavours - "vanillin flavoring affect CFTR activation (altering the salt and water balance at the airway surface) and lead to cell death, compromising the airway epithelium." | Lower |
| ***Cucumber flavours*** | | | | |
| Novelli 2022 | In vitro experimental | ↑ | “In one study, cool cucumber was associated with higher mitochondrial stress, inflammatory cytokines, increased ROS, and barrier dysfunction than other flavors” | Lower |
| ***Banana flavours*** | | | | |
| Novelli 2022 | In vitro experimental | ↑ | “Across several studies, banana pudding and cinnamon demonstrated the most significant alterations in epithelial cell viability [three studies].” | Lower |
| ***Cherry flavours*** | | | | |
| Ward 2020 | Chemical analyses | ↔ | Kośmider et al. [67] detected benzaldehyde in 108 of 145 flavored e-liquids and observed the highest levels (5.1–141.2 μg/30 puffs) in cherry-flavored products | Lower |
| Wilson 2022 | In vitro experimental | ↑ | "Welz et al. tested e-cigarette liquid on oral pharyngeal cells and found apple and cherry flavors caused DNA damage (p < 0.05)." and “significantly reduced cell viability (p < 0.05, p < 0.05, respectively)." | Lower |
| ***Mango flavours*** | | | | |
| Ward 2020 | Chemical analyses | ↔ | One study “analyzed aerosols created from vanilla, cinnamon, and mango e-liquids and observed cadalene in the mango flavor." | Lower |
| ***Citrus flavours*** | | | | |
| Ward 2020 | Chemical analyses | ↑ | One study “generated aerosols from 3 different e-liquids generated by a tank system and estimated freeradical production to be 10.3, 4.0 and 2.5 × 1013 radicals per puff of menthol, citrus, and tobacco flavors, respectively” | Lower |
| Yang 2020 | Human observational | ↑ | “Some e-cigarette users described negative throat symptoms as being associated with particular e-liquid flavors like citrus” | Lower |
| ***Apple flavours*** | | | | |
| Wilson 2022 | In vitro experimental | ↑ | "Welz et al. tested e-cigarette liquid on oral pharyngeal cells and found apple and cherry flavors caused DNA damage (p < 0.05)." and “significantly reduced cell viability (p < 0.05, p < 0.05, respectively)." | Lower |
| ***Velvety flavours*** | | | | |
| Yang 2020 | In vitro experimental | ↑ | "An in vitro study that analyzed the cariogenic potential for various flavored e-cigarette liquids tested five types of flavors (hexyl acetate-apple/plum, ethyl butyrate-pineapple, sucralose-sugar substitute, triacetin- “velvety” or “smoky” flavor, and ethyl maltol-cotton candy). Four out of five flavors (sucralose, ethyl butyrate, triacetin, hexyl acetate) significantly increased biofilm formation compared to an unflavored e-liquid control | Lower |
| ***Smokey flavours*** | | | | |
| Yang 2020 | In vitro experimental | ↑ | "An in vitro study that analyzed the cariogenic potential for various flavored e-cigarette liquids tested five types of flavors (hexyl acetate-apple/plum, ethyl butyrate-pineapple, sucralose-sugar substitute, triacetin- “velvety” or “smoky” flavor, and ethyl maltol-cotton candy). Four out of five flavors (sucralose, ethyl butyrate, triacetin, hexyl acetate) significantly increased biofilm formation compared to an unflavored e-liquid control | Lower |
| ***Buttery/creamy flavours*** | | | | |
| McNeill 2022 | In vitro experimental and animal experimental | ↑ | “Findings from the 13 cell and 9 animal studies suggest there is limited evidence that some flavourings in vaping products, particularly cinnamaldehyde and buttery/creamy flavours, have the potential to alter cellular responses but less than exposure to tobacco smoke. Exposure to unflavoured PG/VG base liquids appeared to have little or no effect.”  “In addition, 23.7% and 31.8% reported having ever used any e-liquid that contained either popcorn flavour/diacetyl or cinnamon flavour/cinnamaldehyde or 2-methoxicinnamaldehyde, respectively. Participants who had ever used these flavours (total n= 154) were more likely than nonusers to report having had a bad reaction (13.0% vs. 2.7%; p < 0.0001).” | Higher |
| ***Pineapple flavours*** | | | | |
| Yang 2020 | In vitro experimental | ↑ | "An in vitro study that analyzed the cariogenic potential for various flavored e-cigarette liquids tested five types of flavors (hexyl acetate-apple/plum, ethyl butyrate-pineapple, sucralose-sugar substitute, triacetin- “velvety” or “smoky” flavor, and ethyl maltol-cotton candy). Four out of five flavors (sucralose, ethyl butyrate, triacetin, hexyl acetate) significantly increased biofilm formation compared to an unflavored e-liquid control | Lower |
| ***Cotton candy flavours*** | | | | |
| Yang 2020 | In vitro experimental | ↑ | "An in vitro study that analyzed the cariogenic potential for various flavored e-cigarette liquids tested five types of flavors (hexyl acetate-apple/plum, ethyl butyrate-pineapple, sucralose-sugar substitute, triacetin- “velvety” or “smoky” flavor, and ethyl maltol-cotton candy). Four out of five flavors (sucralose, ethyl butyrate, triacetin, hexyl acetate) significantly increased biofilm formation compared to an unflavored e-liquid control | Lower |
| ***Grape flavours*** | | | | |
| Yang 2020 | In vitro experimental | ↑ | "Duggar et al. (2018) demonstrated that grape flavor liquid, regardless of nicotine content adversely effected cell growth.” | Lower |
| ***Sour flavours*** | | | | |
| Yang 2020 | Human observational | ↑ | Some e-cigarette users described negative throat symptoms as being associated with particular e-liquid flavors like citrus, sour, cola, custard, or cinnamon (Li et al. 2016; Sinharoy et al. 2018). | Lower |
| ***Cola flavours*** | | | | |
| Yang 2020 | Human observational | ↑ | Some e-cigarette users described negative throat symptoms as being associated with particular e-liquid flavors like citrus, sour, cola, custard, or cinnamon (Li et al. 2016; Sinharoy et al. 2018). | Lower |
| ***Custard flavours*** | | | | |
| Yang 2020 | Human observational | ↑ | Some e-cigarette users described negative throat symptoms as being associated with particular e-liquid flavors like citrus, sour, cola, custard, or cinnamon (Li et al. 2016; Sinharoy et al. 2018). | Lower |

Up arrow is increased potential harm

Equal arrow is no clear evidence of harm

### Supplementary table 5: Harms overlap

|  | **Banks 2023** *(unknown number of studies)* | **Camoni 2023** *(6 studies)* | **Kowitt 2017** *(1 study)* | **Larue 2021** *(17 studies)* | **Lindson 2024** *(3 studies)* | **McNeill 2022** *(3 studies)* | **Notley 2022** *(unknown number of studies)* | **Novelli 2022** *(9 studies)* | **Riley 2016** *(1 study)* | **Ward 2020** *(19 studies)* | **Wilson 2022** *(5 studies)* | **Yang 2020** *(10 studies)* |
| --- | --- | --- | --- | --- | --- | --- | --- | --- | --- | --- | --- | --- |
| **Banks 2023** *(unknown number of studies)* |  | *Unclear* | *Unclear* | *Unclear* | *Unclear* | *Unclear* | *Unclear* | *Unclear* | *Unclear* | *Unclear* | *Unclear* | *Unclear* |
| **Camoni 2023** *(6 studies)* | *Unclear* |  | 0 | 0 | 0 | 0 | *Unclear* | 0 | 0 | 0 | 0 | 0 |
| **Kowitt 2017** *(1 study)* | *Unclear* | 0 |  | 0 | 0 | 0 | *Unclear* | 0 | 0 | 0 | 0 | 0 |
| **Larue 2021** *(17 studies)* | *Unclear* | 0 | 0 |  | 0 | 0 | *Unclear* | 0 | 0 | 0 | 0 | 0 |
| **Lindson 2024** *(3 studies)* | *Unclear* | 0 | 0 | 0 |  | 0 | *Unclear* | 0 | 0 | 0 | 0 | 0 |
| **McNeill 2022** *(3 studies)* | *Unclear* | 0 | 0 | 0 | 0 |  | *Unclear* | 0 | 0 | 0 | 0 | 0 |
| **Notley 2022** *(unknown number of studies)* | *Unclear* | *Unclear* | *Unclear* | *Unclear* | *Unclear* | *Unclear* |  | *Unclear* | *Unclear* | *Unclear* | *Unclear* | *Unclear* |
| **Novelli 2022** *(9 studies)* | *Unclear* | 0 | 0 | 0 | 0 | 0 | *Unclear* |  | 0 | 0 | 0 | 0 |
| **Riley 2016** *(1 study)* | *Unclear* | 0 | 0 | 0 | 0 | 0 | *Unclear* | 0 |  | 0 | 0 | 0 |
| **Ward 2020** *(19 studies)* | *Unclear* | 0 | 0 | 0 | 0 | 0 | *Unclear* | 0 | 0 |  | 0 | 1 |
| **Wilson 2022** *(5 studies)* | *Unclear* | 0 | 0 | 0 | 0 | 0 | *Unclear* | 0 | 0 | 0 |  | 3 |
| **Yang 2020** *(10 studies)* | *Unclear* | 0 | 0 | 0 | 0 | 0 | *Unclear* | 0 | 0 | 1 | 3 |  |

### Supplementary table 6: Vaping uptake overlap

|  | **Dautzenberg 2023** *(4 studies)* | **Han 2022**   *(7 studies)* | **Notley 2022**   *(6 studies)* | **Yoong 2021b**   *(1 study)* |
| --- | --- | --- | --- | --- |
| **Dautzenberg 2023** *(4 studies)* |  | 0 | 0 | 1 |
| **Han 2022**   *(7 studies)* | 0 |  | 2 | 0 |
| **Notley 2022**   *(6 studies)* | 0 | 2 |  | 0 |
| **Yoong 2021b**   *(1 study)* | 1 | 0 | 0 |  |

### Supplementary table 7: Preference, use and/or selection of specific flavors or flavor groups

| **Review ID** | **Flavour types** | **Population** | **Supporting information** | **Review quality** |
| --- | --- | --- | --- | --- |
| Calder 2021 | Sweet/fruit | Pregnant people | "One study that included 16 people who vaped when pregnant was the only study to report flavors used. Among that group, fruit was the most commonly used flavor followed by candy and mint." | Higher |
| Gades 2022 | All |  | “young adults and exclusive e-cigarette users who are either cigarette naïve or recent quitters were more likely to use nontobacco/menthol flavors, while increasing age, dual use, and cigarette only use were more likely to choose tobacco or menthol flavors.” | Lower |
| Feirman 2016 | All | Current and former combustible tobacco users | "One study assessed e-cigarette flavor choice by smoking status and found that current and ex-smokers did not differ with respect to their flavor preferences" Preferred e-cigarette flavours were as follows: (participants could use multiple flavours): tobacco (n=664, 53%), fruit (n=421, 33%), mint/menthol (n=357, 28%), chocolate/sweet flavor (n=231, 18%), coffee (n=167, 13%), other (n=196, 16%), vanilla (n=156, 12%), alcohol related (n=49, 4%) and flavorless (n=11, 1%)  "in one of these new studies, the authors found that the importance of flavoring in e-cigarettes differed by product type; participants using advanced generation devises, as opposed to first generation devices, were more likely to rate having a variety of flavor choices as being important." | Lower |
| Hod 2022 | Sweet/fruit | People aiming to control body weight | "Vanilla and coffee-flavoured e-liquids are among the flavours that were significantly associated with e-cigarette used to control body weight." | Higher |
| Lindson 2024 | All | Combustible tobacco users | “Eleven of the 18 studies offering a choice of flavours provided a breakdown of flavours used at one or more time points. Six of these studies (conducted in the US and UK) showed a higher popularity of sweet over menthol/mint and tobacco flavours, with another study, conducted in Switzerland, finding that 75% of participants used a flavour other than tobacco (sweet or menthol/mint, with no preference breakdown across these categories). The only two studies conducted in Italy both found a preference for tobacco flavour – just over 80% of participants chose tobacco flavour in both (one study offered a choice of tobacco and menthol and the other a choice of tobacco, menthol and sweet). One study (UK-based) observed no clear demarcation in preferences between sweet, tobacco and mint/menthol flavours. The final study reporting on preferences reported that most participants opted for menthol/mint (54%) over tobacco (17.6%) and sweet flavours (28%). This study was carried out in the US and recruited Latinx and African‐American participants, who more commonly smoke menthol cigarettes than the remaining US population. Further to this, Xu 2023 investigated flavour choice moderated by whether participants smoked mentholated or non-mentholated cigarettes on entry into their study. When looking at the study arm where participants were given a choice of flavours (where there was a greater preference for sweet flavours overall), menthol/mint flavours were most popular among participants who usually smoked mentholated cigarettes (52%, compared to 9% choosing tobacco flavours and 39% choosing sweet flavours), whereas tobacco and sweet flavours were similarly popular among participants who usually smoked non-mentholated cigarettes (43% and 46% respectively, compared to 11% menthol/mint flavours)” | Higher |
| Yoong 2021a | All | General | "Three countries (New Zealand, the UK, and the USA) reported on whether participants used flavoured products. In New Zealand, 68% of ever ENDS or ENNDS users reported using a sweet flavour in their last use in the 2018 Youth Insights Survey. Similarly, 63% of current users reported using sweet flavours. In the UK, in ENDS or ENNDS users aged between 11 and 18 years, 45% reported using fruit flavours. The US 2018 National Youth Tobacco Survey (which provides the most recent estimate for flavoured products) found that an estimated 72·2% (95% CI 69·1–75·1) of high school students (aged 14 to 18 years) who were current exclusive users of ENDS or ENNDS used flavoured products. In middle school (aged 11 to 14 years) students in the same survey, 59% (54·8–63·4) reported use of flavoured ENDS or ENNDS. The most frequently reported flavour categories were fruit (high school: 66% [62·4–69·5]; middle school: 68% [62·6–72·5]), and menthol or mint flavour (high school: 57% [53·3–61·3]; middle school: 31% [25·6–37·2]). The US monitoring the future study found that 19% of eighth, tenth, and 12th graders (approximately between 13 years and 18 years) reported using JUUL (Juul Labs, San Francisco, CA, USA), a brand of ENDS and ENNDS, in the past 30 days (since completing the survey). In current users, the flavours used by eighth grade students were mango (33·5%; 95% CI 28·7–38·7), mint (29·2%; 22·7–36·8), and other fruit (16·0%; 12·1–20·9). In tenth graders who are current users, mint (43·5%; 37·1–50·1), mango (27·3%; 23·1–31·9), and other fruit (10·8%; 8·1–14·1) were most popular. In 12th graders who are current users, mint (47·1%; 41·5–52·8), mango (23·8%; 18·8–29·7), and other fruit (8·6%; 6·0–12·0) were also the most popular. | Lower |
| McNeill 2022 | All | Young people | [From a non-systematic synthesis of selected representative surveys]  "In 2021, the most common reasons for vaping reported by young people were to ‘give it a try’ (2021 ASH-Y– 48.8%) or ‘curiosity/to try something new’ (ITC – 20.5%), and ‘for the flavours’ (ITC – 37.2%) or ‘other people use them so I join in’ (2021 ASH-Y– 16.6%)."  "The 2021 ASH-Y data estimated that fruit flavoured vaping products were used by 51.5% of 11 to 18 year olds who vaped (Figure 12) followed by menthol or mint (13.0%) and chocolate, desserts, sweet or candy flavours (9.3%). These flavour patterns are very similar to the ASH-Y 2020 data used in our 2021 report, where fruit was the most commonly used (46.3%), followed by menthol or mint (18.1%) and chocolate, sweets or candy (8.5%). The ITC Youth data reports similar flavours that were most often used among 16 to 19 year olds who vaped in the last 30 days, with 63.7% using fruit flavours, 27.7% using menthol or mint flavours and 10.6% using chocolate, desserts, sweet or candy flavours (Figure 13). The ITC Youth survey also indicates that 11.1% used tobacco flavours (0.6% in ASH-Y). All other flavours, across both surveys, were used by less than 10% of included participants. Again, these flavour patterns are very similar to the ITC Youth 2019 data used in our 2021 report, where fruit was the most commonly used (67.7%), followed by menthol or mint (18.3%), chocolate, sweets or candy (13.5%) and tobacco (10.3%)." | Higher |
|  | All | Adults | [From a non-systematic synthesis of selected representative surveys]  "The 2021 ASH-A survey asked people who vaped about the flavour they used most often. Fruit flavours were the main flavour for 35.3% of current vapers, menthol/mint flavours for 22.5% and tobacco flavours for 20.9%. All other flavours were used by less than 10% of current vapers—a pattern which aligns with our previous report. When broken down by socio-demographics (table 15), tobacco flavours seemed to be preferred by older vapers, with 35.6% of vapers aged over 55 using tobacco-flavoured vaping liquids. Among younger vapers, around half of 18 to 24 year olds (49.7%) and 25 to 34 year olds (50.5%) preferred fruit flavours. More female vapers (27.4%) preferred menthol/mint flavours than male vapers (18.6%), but the difference was not statistically significant (χ2 = 6.0, p=0.051). There were no discernible differences in flavour preferences by region, social grade or ethnicity. Despite the small sample size, it is worth noting that never smokers who vaped almost exclusively reported fruit flavours as their preferred flavour." |  |

### Supplementary table 8: Combustible tobacco cessation/reduction

|  | **Ciapponi 2021** *(1 study)* | **Liber 2023** *(29 studies)* | **Lindson 2024** *(19 studies)* | **Meernik 2019** *(9 studies)* | **Notley 2022** *(2 studies)* | **Yoong 2021b** *(1 study)* |
| --- | --- | --- | --- | --- | --- | --- |
| **Ciapponi 2021** *(1 study)* |  | 1 | 0 | 1 | 0 | 0 |
| **Liber 2023** *(29 studies)* | 1 |  | 0 | 4 | 1 | 1 |
| **Lindson 2024** *(19 studies)* | 0 | 0 |  | 0 | 0 | 0 |
| **Meernik 2019** *(9 studies)* | 1 | 4 | 0 |  | 0 | 0 |
| **Notley 2022** *(2 studies)* | 0 | 1 | 0 | 0 |  | 1 |
| **Yoong 2021b** *(1 study)* | 0 | 1 | 0 | 0 | 1 |  |

### Supplementary table 9: Combustible tobacco initiation

|  | **Notley 2022** *(2 studies)* | **Yoong 2021b** *(1 study)* |
| --- | --- | --- |
| **Notley 2022** *(2 studies)* |  | 1 |
| **Yoong 2021b** *(1 study)* | 1 |  |

### Supplementary table 10: Excluded studies

| **Reference** | **Reason for exclusion** |
| --- | --- |
| Akiyama Y, Sherwood N. Systematic review of biomarker findings from clinical studies of electronic cigarettes and heated tobacco products. Toxicology Reports. 2021; 8: 282-294.DOI: 10.1016/j.toxrep.2021.01.014 | Does not look at flavors |
| Anandan, Aathavan Shanmuga; Leung, Janni; Chan, Gary C. K; Sun, Tianze; Connor, Jason P; Hall, Wayne D; Stjepanovic, Daniel. Common adverse events of electronic cigarettes compared with traditional nicotine replacement therapies: A systematic review and meta-analysis. Drug and Alcohol Review . 2023;42(5):1278-1287. <https://doi.org/10.1111/dar.13674> | Does not look at flavors |
| Armendariz-Castillo, Isaac; Guerrero, Santiago; Vera-Guapi, Antonella; Cevallos-Vilatuna, Tiffany; Garcia-Cardenas, Jennyfer M; Guevara-Ramirez, Patricia; Lopez-Cortes, Andres; Perez-Villa, Andy; Yumiceba, Veronica; Zambrano, Ana K; Leone, Paola E; Paz-Y-Mino, Cesar. Genotoxic and Carcinogenic Potential of Compounds Associated with Electronic Cigarettes: A Systematic Review. BioMed research international / 2019;2019(101600173):1386710. <https://doi.org/10.1155/2019/1386710> | Does not look at flavors |
| Baniulyte, G., Ali, K. E-cigarette side effects in otolaryngology: unveiling the vape mirage. *Evid Based Dent* 24, 184–185 (2023). <https://doi.org/10.1038/s41432-023-00941-0> | Does not look at flavors |
| Barufaldi LA, Guerra RL, de Albuquerque R D CR, Nascimento A, Chança RD, de Souza MC et al. Risk of smoking relapse with the use of electronic cigarettes: A systematic review with meta-analysis of longitudinal studies. Tobacco Prevention & Cessation. 2021;7(April):29. <https://doi.org/10.18332/tpc/132964> | Does not look at flavors |
| Timothy D Becker, Melanie K Arnold, Vicky Ro, Lily Martin, Timothy R Rice, Systematic Review of Electronic Cigarette Use (Vaping) and Mental Health Comorbidity Among Adolescents and Young Adults, *Nicotine & Tobacco Research*, Volume 23, Issue 3, March 2021, Pages 415-425, <https://doi.org/10.1093/ntr/ntaa171> | Does not look at flavors |
| Bell, L., Whelan, M., Thomas, L. et al. Use of e-cigarettes in pregnancy: A systematic review of evidence published from 2020-2022. J Public Health (Berl.) (2023). <https://doi.org/10.1007/s10389-023-02026-9> | Does not look at flavors |
| Bernat JK, Jackson KJ, Krüsemann EJZ*, et al.* Sensory methods to evaluate perception of flavours in tobacco and other nicotine-containing products: a review. *Tobacco Control* 2023;**32:**e95-e102. <https://doi.org/10.1136/tobaccocontrol-2021-056681> | Does not look at flavors |
| Bjurlin, Marc A; Matulewicz, Richard S; Roberts, Timothy R; Dearing, Bianca A; Schatz, Daniel; Sherman, Scott; Gordon, Terry; Shahawy, Omar El. Carcinogen Biomarkers in the Urine of Electronic Cigarette Users and Implications for the Development of Bladder Cancer: A Systematic Review. European urology oncology. 2021;4(5):766-783. DOI:10.1016/j.euo.2020.02.004 | Does not look at flavors |
| Bourke, Liam; Bauld, Linda; Bullen, Christopher; Cumberbatch, Marcus; Giovannucci, Edward; Islami, Farhad; McRobbie, Hayden; Silverman, Debra T; Catto, James W F. E-cigarettes and Urologic Health: A Collaborative Review of Toxicology, Epidemiology, and Potential Risks. European urology / 2017;71(6):915-923. DOI:10.1016/j.eururo.2016.12.022 | Does not look at flavors |
| Burstyn, I. Peering through the mist: systematic review of what the chemistry of contaminants in electronic cigarettes tells us about health risks. BMC Public Health 14, 18 (2014). <https://doi.org/10.1186/1471-2458-14-18> | Does not look at flavors |
| Butler, Ailsa R; Lindson, Nicola; Fanshawe, Thomas R; Theodoulou, Annika; Begh, Rachna; Hajek, Peter; McRobbie, Hayden; Bullen, Chris; Notley, Caitlin; Rigotti, Nancy A; Hartmann-Boyce, Jamie. Longer-term use of electronic cigarettes when provided as a stop smoking aid: Systematic review with meta-analyses. Preventive Medicine: An International Journal Devoted to Practice and Theory / 2022;165(Part B):1-12. DOI: 10.1016/j.ypmed.2022.107182 | Does not look at flavors |
| Glasser AM, Cobb CO, Teplitskaya L*, et al* Electronic nicotine delivery devices, and their impact on health and patterns of tobacco use: a systematic review protocol. *BMJ Open* 2015;**5:**e007688. doi: 10.1136/bmjopen-2015-007688 | Wrong study design |
| Hartmann-Boyce J, Begh R, Lindson N, Livingstone-Banks J, Fanshawe TR, McNeill A, Shahab L, Rigotti NA, Kneale D, Thomas J, Aveyard P. Electronic cigarettes and subsequent cigarette smoking in young people. Cochrane Database of Systematic Reviews 2022, Issue 3. Art. No.: CD015170. DOI: 10.1002/14651858.CD015170 | Does not look at flavors |
| Hartmann-Boyce J, Butler AR, Theodoulou A, Onakpoya IJ, Hajek P, Bullen C, et al. Biomarkers of potential harm in people switching from smoking tobacco to exclusive e-cigarette use, dual use or abstinence: secondary analysis of Cochrane systematic review of trials of e-cigarettes for smoking cessation. Addiction. 2023; 118(3): 539–545. <https://doi.org/10.1111/add.16063> | Does not look at flavors |
| Hess, Isabel Mr; Lachireddy, Kishen; Capon, Adam. A systematic review of the health risks from passive exposure to electronic cigarette vapour. Public health research & practice / 2016;26(2). DOI: [10.17061/phrp2621617](https://dx.doi.org/10.17061/phrp2621617) | Does not look at flavors |
| Hexin Katengeza, Liling Huang, Roselyn Chipojola, Chieh-Feng Chen, Jiun-Hua Huang, Usman Iqbal. Impact of non-menthol flavors in tobacco products on perception and use among youth, young adults and adults: an updated systematic review and meta-analysis. CRD42020166841 | No published review |
| Hoffman AC, Salgado RV, Dresler C, et al. Flavour preferences in youth versus adults: a review. Tobacco Control 2016;25:ii32-ii39. | Wrong study design |
| Honeycutt, L., Huerne, K., Miller, A. et al. A systematic review of the effects of e-cigarette use on lung function. npj Prim. Care Respir. Med. 32, 45 (2022). <https://doi.org/10.1038/s41533-022-00311-w> | Does not look at flavors |
| Erna J Z Krüsemann, Sanne Boesveldt, Kees de Graaf, Reinskje Talhout, An E-Liquid Flavor Wheel: A Shared Vocabulary Based on Systematically Reviewing E-Liquid Flavor Classifications in Literature, Nicotine & Tobacco Research, Volume 21, Issue 10, October 2019, Pages 1310–1319, <https://doi.org/10.1093/ntr/nty101> | Wrong outcomes |
| J Y Levett, K B Filion, P Reynier, C Prell, M J Eisenberg, Efficacy and safety of e-cigarette use for smoking cessation: a systematic review and meta-analysis of randomized controlled trials, European Heart Journal, Volume 42, Issue Supplement_1, October 2021, ehab724.2600, <https://doi.org/10.1093/eurheartj/ehab724.2600> | Does not look at flavors |
| Liu, Xing MDa,b; Lu, Wan MDc; Liao, Sheng MDd; Deng, Zhongliang MDb; Zhang, Zhongrong MDd; Liu, Yun MDe,*; Lu, Weizhong MDa,*. Efficiency and adverse events of electronic cigarettes: A systematic review and meta-analysis (PRISMA-compliant article). Medicine 97(19):p e0324, May 2018. \| DOI: 10.1097/MD.0000000000010324 | Does not look at flavors |
| Martinez-Morata, I., Sanchez, T.R., Shimbo, D. et al. Electronic Cigarette Use and Blood Pressure Endpoints: a Systematic Review. Curr Hypertens Rep 23, 2 (2021). <https://doi.org/10.1007/s11906-020-01119-0> | Wrong study design |
| Xing-chen Meng, Xin-xin Guo, Zhen-yan Peng, Chun Wang, Ran Liu, Acute effects of electronic cigarettes on vascular endothelial function: a systematic review and meta-analysis of randomized controlled trials, European Journal of Preventive Cardiology, Volume 30, Issue 5, April 2023, Pages 425–435, <https://doi.org/10.1093/eurjpc/zwac248> | Does not look at flavors |
| Pisinger, Charlotta; Dossing, Martin. A systematic review of health effects of electronic cigarettes. Preventive medicine / 2014;69(pm4, 0322116):248-60. DOI: [10.1016/j.ypmed.2014.10.009](https://dx.doi.org/10.1016/j.ypmed.2014.10.009) | Wrong study design |
| Pisinger C, Rasmussen SKB. The Health Effects of Real-World Dual Use of Electronic and Conventional Cigarettes versus the Health Effects of Exclusive Smoking of Conventional Cigarettes: A Systematic Review. International Journal of Environmental Research and Public Health. 2022; 19(20):13687. <https://doi.org/10.3390/ijerph192013687> | Does not look at flavors |
| Pound CM, Zhang JZ, Kodua AT, et alSmoking cessation in individuals who use vaping as compared with traditional nicotine replacement therapies: a systematic review and meta-analysisBMJ Open 2021;11:e044222. doi: 10.1136/bmjopen-2020-044222 | Does not look at flavors |
| Qureshi, M.A., Vernooij, R.W.M., La Rosa, G.R.M. et al. Respiratory health effects of e-cigarette substitution for tobacco cigarettes: a systematic review. Harm Reduct J 20, 143 (2023). <https://doi.org/10.1186/s12954-023-00877-9> | Does not look at flavors |
| Salam, Sally; Saliba, Najat Aoun; Shihadeh, Alan; Eissenberg, Thomas; El-Hellani, Ahmad. Flavor-Toxicant Correlation in E-cigarettes: A Meta-Analysis. Chemical research in toxicology / 2020;33(12):2932-2938. DOI: 10.1021/acs.chemrestox.0c00247 | Wrong outcomes |
| Shi, Molin, Jordan A. Gette, Tre D. Gissandaner, Jeffrey T. Cooke, and Andrew K. Littlefield. 2020. “E-Cigarette Use among Asian Americans: A Systematic Review.” Journal of Ethnicity in Substance Abuse 21 (4): 1165–98. doi:10.1080/15332640.2020.1861495. | Does not look at flavors |
| Siddiqi, Tariq Jamal; Rashid, Ahmed Mustafa; Siddiqi, Ahmed Kamal; Anwer, Anusha; Usman, Muhammad Shariq; Sakhi, Hifza; Bhatnagar, Aruni; Hamburg, Naomi M; Hirsch, Glenn A; Rodriguez, Carlos J; Blaha, Michael J; DeFilippis, Andrew P; Benjamin, Emelia J; Hall, Michael E. Association of Electronic Cigarette Exposure on Cardiovascular Health: A Systematic Review and Meta-Analysis. Current problems in cardiology / 2023;48(9):101748. DOI: 10.1016/j.cpcardiol.2023.101748 | Does not look at flavors |
| Soneji S, Barrington-Trimis JL, Wills TA, et al. Association Between Initial Use of e-Cigarettes and Subsequent Cigarette Smoking Among Adolescents and Young Adults: A Systematic Review and Meta-analysis. JAMA Pediatr. 2017;171(8):788–797. doi:10.1001/jamapediatrics.2017.1488 | Does not look at flavors |
| Sreedharan, Subhashaan et al. Radiological findings of e-cigarette or vaping product use associated lung injury: A systematic review.Heart & Lung: The Journal of Cardiopulmonary and Acute Care, Volume 50, Issue 5, 736 - 741 | Does not look at flavors |
| Szumilas K, Szumilas P, Grzywacz A, Wilk A. The Effects of E-Cigarette Vapor Components on the Morphology and Function of the Male and Female Reproductive Systems: A Systematic Review. International Journal of Environmental Research and Public Health. 2020; 17(17):6152. <https://doi.org/10.3390/ijerph17176152> | Wrong study design |
| Thomas KH, Dalili MN, López-López JA, Keeney E, Phillippo DM, Munafò MR, et al. Comparative clinical effectiveness and safety of tobacco cessation pharmacotherapies and electronic cigarettes: a systematic review and network meta-analysis of randomized controlled trials. Addiction. 2022; 117: 861–876. <https://doi.org/10.1111/add.15675> | Does not look at flavors |
| Thomas KH, Dalili MN, López-López JA, Keeney E, Phillippo D, Munafò MR, et al. Smoking cessation medicines and e-cigarettes: a systematic review, network meta-analysis and cost-effectiveness analysis. Health Technol Assess 2021;25(59). <https://doi.org/10.3310/hta25590> | Does not look at flavors |
| Vanderkam, Paul; Bonneau, Audrey; Kinouani, Sherazade; Dzeraviashka, Palina; Castera, Philippe; Besnier, Marc; Binder, Philippe; Doux, Nicolas; Jaafari, Nematollah; Lafay-Chebassier, Claire. Duration of the effectiveness of nicotine electronic cigarettes on smoking cessation and reduction: Systematic review and meta-analysis. Frontiers in psychiatry / 2022;13(101545006):915946. DOI: [10.3389/fpsyt.2022.915946](https://dx.doi.org/10.3389/fpsyt.2022.915946) | Does not look at flavors |
| Vu GT, Stjepanović D, Sun T, et alPredicting the long-term effects of electronic cigarette use on population health: a systematic review of modelling studiesTobacco Control Published Online First: 09 June 2023. doi: 10.1136/tc-2022-057748 | Does not look at flavors |
| Wasfi, Rania A; Bang, Felix; de Groh, Margaret; Champagne, Andre; Han, Arum; Lang, Justin J; McFaull, Steven R; Melvin, Alexandria; Pipe, Andrew Lawrence; Saxena, Shika; Thompson, Wendy; Warner, Emily; Prince, Stephanie A. Chronic health effects associated with electronic cigarette use: A systematic review. Frontiers in public health / 2022;10(101616579):959622. DOI: [10.3389/fpubh.2022.959622](https://dx.doi.org/10.3389/fpubh.2022.959622) | Does not look at flavors |
| Xantus, Gabor; Anna Gyarmathy, Valeria; Johnson, Carole Ann; Sanghera, Pavanjit; Zavori, Laszlo; Kanizsai, Peter Laszlo. The role of vitamin E acetate (VEA) and its derivatives in the vaping associated lung injury: systematic review of evidence. Critical reviews in toxicology / 2021;51(1):15-23 | Wrong intervention |
| Yayan, Josef; Franke, Karl-Josef; Biancosino, Christian; Rasche, Kurt. Comparative systematic review on the safety of e-cigarettes and conventional cigarettes. British Industrial Biological Research Association / 2024;185(f3u, 8207483):114507. | Does not look at flavors |
| Zakiyah, Neily; Purwadi, Febby V; Insani, Widya N; Abdulah, Rizky; Puspitasari, Irma M; Barliana, Melisa I; Lesmana, Ronny; Amaliya, Amaliya; Suwantika, Auliya A. Effectiveness and Safety Profile of Alternative Tobacco and Nicotine Products for Smoking Reduction and Cessation: A Systematic Review. Journal of multidisciplinary healthcare / 2021;14(101512691):1955-1975. | Does not look at flavors |
| Zare, Samane; Nemati, Mehdi; Zheng, Yuqing. A systematic review of consumer preference for e-cigarette attributes: Flavor, nicotine strength, and type. PLoS ONE / 2018;13(3) | Wrong study design |
| Zatoński M, Silver K, Plummer S, Hiscock R. Menthol and flavored tobacco products in LMICs: A growing menace. Tobacco Induced Diseases. 2022;20(April):39. <https://doi.org/10.18332/tid/146366> | Wrong intervention |
| Zepeta-Hernandez, David; Armendariz-Garcia, Nora Angelica; Martinez-Diaz, Nazaria; Alonso-Castillo, Maria Magdalena. [Uso de cigarros electronicos en los espacios libres de humo y vapeo: una revision sistematica.] Revista espanola de salud publica / 2023;97 | Does not look at flavors |
| Zhang Y, Bu F, Dong F, Wang J, Zhu S, Zhang X et al. The effect of e-cigarettes on smoking cessation and cigarette smoking initiation: An evidence-based rapid review and meta-analysis. Tobacco Induced Diseases. 2021;19(January):4. <https://doi.org/10.18332/tid/131624> | Does not look at flavors |
| Zhao, Di; Aravindakshan, Atul; Hilpert, Markus; Olmedo, Pablo; Rule, Ana M; Navas-Acien, Ana; Aherrera, Angela. Metal/Metalloid Levels in Electronic Cigarette Liquids, Aerosols, and Human Biosamples: A Systematic Review. Environmental health perspectives / 2020;128(3):36001 | Does not look at flavors |
| Zhao+ K, Li+ J, Zhou P, Xu L, Yang M. Is electronic cigarette use a risk factor for stroke? A systematic review and meta-analysis. Tobacco Induced Diseases. 2022;20(November):101. <https://doi.org/10.18332/tid/154364> | Does not look at flavors |
| Zhong J, Cao S, Gong W, Fei F, Wang M. Electronic Cigarettes Use and Intention to Cigarette Smoking among Never-Smoking Adolescents and Young Adults: A Meta-Analysis. International Journal of Environmental Research and Public Health. 2016; 13(5):465. <https://doi.org/10.3390/ijerph13050465> | Does not look at flavors |
| Zulkifli A, Abidin E, Abidin N, Amer Nordin A, Praveena S, Syed Ismail S, Rasdi I, Karuppiah K, Rahman A. Electronic cigarettes: a systematic review of available studies on health risk assessment. Reviews on Environmental Health. 2018;33(1): 43-52. <https://doi.org/10.1515/reveh-2015-0075> | Wrong study design |

### Search strategy for Ovid databases (MEDLINE, Embase, PsycINFO)

1. e-cig*.mp. OR ecig*.mp. OR electr* cigar*.mp. OR electronic nicotine.mp. OR (vape or vapes or vaporizer or vapourizer or vaporiser or vapouriser or vaper or vapers or vaping).ti,ab. OR Exp Electronic Nicotine Delivery Systems/
2. (flavor* OR flavour*).mp OR flavoring agents/
3. Meta-Analysis as Topic/ OR meta analy$.tw. OR metaanaly$.tw. OR Meta-Analysis/ OR (systematic adj (review$1 or overview$1)).tw. OR evidence synthesis.tw. OR exp Review Literature as Topic/
4. cochrane.ab. OR embase.ab. OR (psychlit or psyclit).ab. OR (psychinfo or psycinfo).ab. OR (cinahl or cinhal).ab. OR science citation index.ab. OR bids.ab. OR cancerlit.ab.
5. reference list$.ab. OR bibliograph$.ab. OR hand-search$.ab. OR relevant journals.ab. OR manual search$.ab.
6. (selection criteria.ab. OR data extraction.ab.) AND Review/
7. 3 OR 5 OR 6
8. Comment/ OR Letter/ OR Editorial/ OR (animal/ NOT (animal/ AND human/))
9. 7 NOT 8
10. 1 AND 2 AND 9
